# Taxonomic revision of *Pachyptera* (Bignonieae, Bignoniaceae)

**DOI:** 10.3897/phytokeys.92.20987

**Published:** 2018-01-19

**Authors:** Jessica Nayara Carvalho Francisco, Lúcia G. Lohmann

**Affiliations:** 1 Universidade de São Paulo, Departamento de Botânica, Rua do Matão, 277, 05508-090, São Paulo, SP, Brazil

**Keywords:** Amazon, Flora, *Pachyptera kerere*, Neotropics, Taxonomy

## Abstract

*Pachyptera* DC. is a small genus of neotropical lianas included in tribe Bignonieae (Bignoniaceae). The genus has a complicated taxonomic history but currently includes species distributed from Belize to Southern Amazon. *Pachyptera* is characterised by four main synapomorphies, namely, a papery peeling bark, prophylls of the axillary buds organised in a series of three, patelliform glands arranged in lines in the upper portions of the calyx and corolla tube. Furthermore, members of the genus also have stems with four phloem wedges in cross-section and conspicuous extrafloral nectaries between the interpetiolar region and at the petiole apex, although these characters are also shared with other genera of tribe Bignonieae. Here, we present a taxonomic revision of *Pachyptera*, which includes a complete list of synonyms, detailed morphological descriptions of species and an identification key, as well as information on the habitat, distribution and phenology, nomenclatural notes, taxonomic comments and illustrations of all the species. In addition, we designate three lectotypes, propose one new combination, raise one variety to species status and describe a new species. After these adjustments, a *Pachyptera* with five well-defined species is recognised.

## Introduction

*Pachyptera* DC. (Bignonieae, Bignoniaceae) includes neotropical lianas that are distributed from Belize to central Brazilian Amazon, with most species restricted to wet Amazonian forests (Lohmann and Taylor 2014). The genus has a complicated taxonomic history, including a difficult generic circumscription and several poorly defined taxa. Here, a new systematic treatment of *Pachyptera* is proposed and five species are recognised using an integrative approach that includes data derived from a recent molecular phylogeny (Francisco and Lohmann 2017, Francisco and Lohmann submitted), coalescent approaches (Francisco and Lohmann submitted) and new morphological studies.

This new classification recognises a monophyletic genus that is characterised by four morphological synapomorphies, namely, a papery peeling bark, prophylls of the axillary buds organised in a series of three (Lohmann and Taylor 2014), patteliform glands arranged in lines on the upper portions of the calyx and corolla tube (Lohmann 2006, Lohmann and Taylor 2014). In addition, stems with four phloem wedges in cross-section, conspicuous extrafloral nectaries on the interpetiolar region and at the petiole apex also help to identify members of the genus (Lohmann and Taylor 2014).

## Taxonomic history

*Pachyptera* was originally described by de Candolle (1845), who characterised the genus by a compressed capsule and seeds with coriaceous wings. The genus originally included six species, four of which [i.e. *P. umbelliformis* DC., *P. striata* DC., *P. dasyantha* DC. and *P. perrottetii* DC.] are synonyms of *Tanaecium pyramidatum* (Rich.) L.G. Lohmann, while *P. puberula* DC. is a synonym of *Dolichandra uncata* (Andrews) L.G. Lohmann. Only *P. foveolata* DC. remains in *Pachyptera*, although as a synonym of *Pachyptera kerere* (Aubl.) Sandwith.

Nearly five decades after being described, *Pachyptera* was synonymised into *Adenocalymma* Mart. ex Meisn by Baillon (1891) based on the broad and thick capsule shared amongst members of these genera. Subsequently, Bureau and Schumann (1896 [1897]) transferred *P. foveolata* to Adenocalymma section Pachyptera, which was characterised by villous anthers and plate-shaped glands arranged in lines outside the corolla tube, right below the lobes. At the same time, *P. kerere* was transferred to Adenocalymma section Hanburyophyton together with four species of *Mansoa*, i.e. *A. alliaceum* (Lam.) Miers, *A. asperulum* Bureau & K. Schum., *A. splendens* Bureau & Schum. [= *Mansoa difficilis*] and *A. lanceolatum* Miers. *Pachyptera* was subsequently segregated from *Adenocalymma* by Sprague and Sandwith (1932) and restored to generic rank, as a monotypic genus that only included *P. foveolata*.

*Pachyptera foveolata*, as circumscribed by Sprague and Sandwith (1932), consisted of a species complex that included individuals with white to crimson flowers. While the authors themselves recognised the difficulties associated with the recognition of such a diverse species, the restricted sampling prevented them from analysing the breadth of morphological variation included in this group and the recognition of a single species. Five years later, Sandwith (1937) noted that Aublet’s epithet “*kerere*” was the correct name for *P. foveolata* and proposed the new combination *Pachyptera kerere*. Dugand (1955) also noted the high variation found in flower traits of specimens of *Pachyptera kerere* and described the new variety P. kerere var. erythraea Dugand. The variety P. kerere var. erythraea differs from P. kerere var. kerere in the red corolla (vs. white in P. kerere var. kerere). Gentry (1977) subsequently noted that P. kerere var. kerere and P. kerere var. erythraea also differed in the sub-exserted to exserted anthers (vs. included anthers in P. kerere var. kerere), campanulate corolla with 11–15 mm in diameter (vs. sub-bilabiate corolla with 3–7 mm in diameter in P. kerere var. kerere) and leaf blade puberulous (vs. leaf blade glabrous in P. kerere var. kerere), which led him to raise *P. erythraea* (Dugand) A.H. Gentry to species rank.

Although *Bignonia incarnata* Aubl. was described in the same work as *Bignonia kerere* Aubl. (1775), the close relationship between those two taxa was not noted. In fact, *Bignonia incarnata* was thought to be morphologically similar and perhaps more closely related to *Cydista aequinoctialis* (L.) Miers by various authors (see Sandwith 1937). Nearly two decades later, Gentry (1973) noted the similarity between individuals of *B. incarnata* and *P. kerere*, which led him to treat *B. incarnata* as a variety of *P. kerere*, i.e. Pachyptera kerere var. incarnata (Aubl.) A.H. Gentry. At the same time, Gentry (1973) reduced *Pseudocalymma* [= *Mansoa*] into *Pachyptera* due to the shared trifid tendrils, white to red or purple flowers, interpetiolar gland-fields, 3-colpate pollen and deciduous bracts. In this work, three species of *Pseudocalymma* were transferred to *Pachyptera* [*P. alliacea* (Lam.) A.H. Gentry, *P. hymenaea* (DC.) A.H. Gentry, and *P. standleyi* (Steyerm.) A.H. Gentry], all of which are currently placed in *Mansoa*.

Gentry (1979) and Gentry and Tomb (1979) used new palynological data as a basis to merge *Pachyptera* and *Hanburyphython* Bureau ex Warm. in *Mansoa* DC., resulting in seven new combinations: *M. alliacea* (Lam.) A.H. Gentry, *M. erythraea* (Dugand) A.H. Gentry, *M. hymenaea* (DC.) A.H. Gentry, M. kerere var. kerere (Aubl.) A.H. Gentry, M. kerere var. incarnata (Aubl.) A.H. Gentry, *M. parvifolia* (A.H. Gentry) A.H. Gentry, and *M. standleyi* (Steyerm.) A.H. Gentry. In addition, a new species was described, *Mansoa ventricosa* A.H. Gentry, a taxon known from the type specimen plus one additional material whose placement was uncertain.

### Phylogenetic based classifications of *Pachyptera*

While the taxonomic confusion between *Mansoa* and *Pachyptera* remained for several years, molecular phylogenetic data (Lohmann 2006) indicated that *Mansoa* and *Pachyptera* are distantly related, while the monotypic *Leucocalantha* Barbosa Rodrigues is closely related to *Pachyptera*. *Leucocalantha* was described based on the long and white corollas that resembled the Asian genus *Millingtonia* L.f. (Oroxyleae, Bignoniaceae). The genus only included *Leucocalantha aromatica* Barb. Rodr., which is characterised by white, pubescent and hypocrateriform corolla tubes and glands at the apices of the petioles and corollas. While the close relationship between *Leucocalantha* and *Pachyptera* was initially surprising, a careful morphological study recovered multiple morphological features shared between these taxa (e.g. stems with four phloem wedges in cross-section, corollas with glands arranged in lines on the upper portions of the tube and racemose inflorescences). This observation led to the re-establishment of *Pachyptera* and the inclusion of *Leucocalantha* into *Pachyptera* in a revised generic classification of the whole tribe Bignonieae (Lohmann and Taylor 2014). Under this classification, *Pachyptera* included four species, i.e. *P. aromatica* (Barb. Rodr.) L.G. Lohmann, *P. erythraea* (Dugand) A.H. Gentry, *P. kerere* and *P. ventricosa* (A.H. Gentry) L.G. Lohmann. This circumscription was based on morphological observations for all taxa and a molecular phylogenetic framework of the whole tribe Bignonieae that sampled half of the species of *Pachyptera* (Lohmann 2006); two rare and morphologically complicated species (i.e. *P. erythraea* and *P. ventricosa*) were not sampled in the phylogeny, raising their generic placement into question.

A recent phylogenetic and morphological study of *Pachyptera* (Francisco and Lohmann 2017) sampled all species recognised by Lohmann and Taylor (2014). In this phylogeny, *Pachyptera ventricosa* was more closely related to *Mansoa* than to other species of *Pachyptera*, which led to the reestablishment of *Mansoa ventricosa* (Francisco and Lohmann 2017). In addition, this study also provided further support for the inclusion of *P. aromatica* and *P. erythraea* into *Pachyptera*. As such, *Pachyptera* was recognised as a monophyletic genus comprising three species. However, the infra-specific classification of the *P. kerere* species complex remained uncertain. More specifically, it remained dubious whether *P. erythraea* and P. kerere var. incarnata should be treated as separate species or varieties of *P. kerere*.

**Figure 1.**
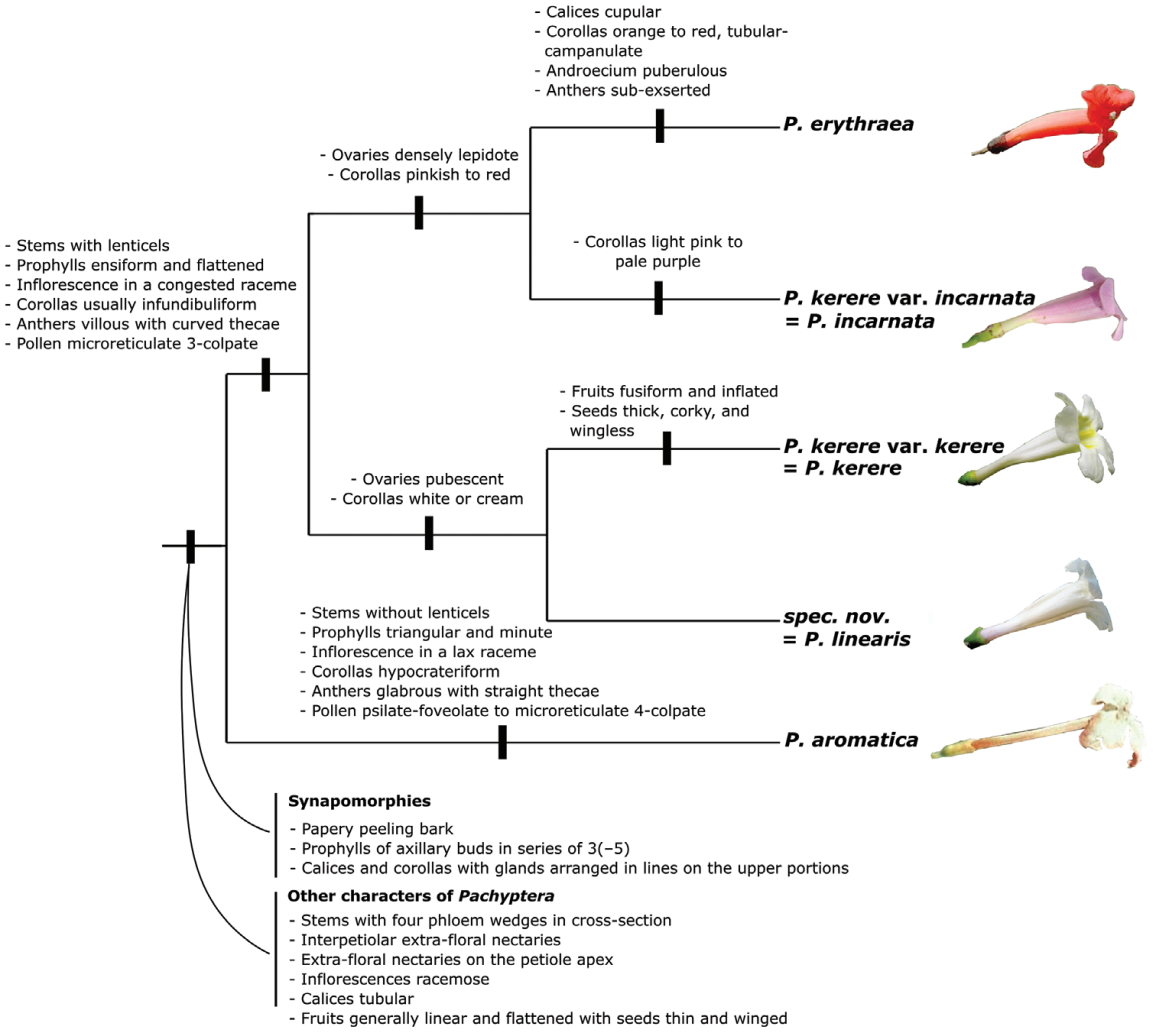
Schematic diagram summarising phylogenetic relationships within *Pachyptera*, with morphological characters mapped on the diagram. Names of the terminal taxa indicate the taxon in which these species were previously included. The taxonomic updates proposed here are also indicated. Relationships depicted follow Francisco and Lohmann (submitted).

A more comprehensive phylogenetic study of the genus (Francisco and Lohmann submitted) sampled multiple individuals of all three species of *Pachyptera* recognised by Francisco and Lohmann (2017) and used coalescent approaches to verify species limits. This study identified five evolutionary units that are characterised by distinct morphological features and ecological traits (Fig. 1). Within this phylogenetic framework, *P. aromatica* is sister to the remaining species of the genus. This species is characterised by a series of morphological autapomorphies such as stems without lenticels, prophylls of axillary buds triangular and minute, inflorescence in lax racemes, corolla hypocrateriform, anthers glabrous with straight thecae and pollen glabrous psilate-foveolate to microreticulate 4-colpate. The remaining species of the genus are divided into two main clades, the first of which includes *P. erythraea* and P. kerere var. incarnata (= *P. incarnata*), both of which are characterised by pinkish to red corollas, with ovaries densely lepidote. *Pachyptera incarnata* is easily distinguished by the light pink to pale purple corollas (vs. orange to red in *P. erythraea*), calyx tubular (vs. cupular in *P. erythraea*), corolla infundibuliform (vs. corolla tubular-campanulate in *P. erythraea*), androecium glabrous (vs. androecium puberulous in *P. erythraea*), anthers included (vs. anthers sub-exserted in *P. erythraea*) and ovaries not-bisulcate (vs. bisulcate in *P. erythraea*). The second clade includes P. kerere var. kerere (= *P. kerere*) and a new species described here (*P. linearis* Francisco & L.G. Lohmann), both characterised by white to cream coloured corollas, with ovaries pubescent (Fig. 1). *Pachyptera kerere* is separated from *P. linearis* by the fruit fusiform (vs. linear in *P. linearis*) and the seeds inflated, thick, corky and wingless (vs. seeds flattened, thin, membranaceous and winged in *P. linearis*).

### Distribution

Almost all *Pachyptera* species are found in the Amazon rainforest. Only *P. kerere* is widely distributed while all other species have restricted distributions. *Pachyptera kerere* is frequent throughout the Amazon and also distributed in Central America from Panama to Belize. *Pachyptera aromatica* is restricted to the Brazilian Amazon. *Pachyptera erythraea* is endemic to the middle Magdalena River Valley of Colombia, while *P. incarnata* is endemic to Eastern Amazon. *Pachyptera linearis* is known only by a few collections from Venezuela and Colombia.

### Habitats

Species of *Pachyptera* occur in wet *terra-firme* forests, generally close to water bodies or riverbanks and are also found in flooded forests such as the Brazilian *igapó*. The majority of *Pachyptera* species has seeds with thin wings that are wind dispersed, while *P. kerere* has thick, corky and wingless seed adapted to water dispersal (Gentry 1976). Water dispersal arose several times independently in tribe Bignonieae, mostly from wind-dispersed ancestors (Gentry 1983, 1990, Lohmann 2004). Shifts between dispersal syndromes may have driven the speciation of *P. kerere* (Francisco and Lohmann submitted).

### Reproductive biology

*Pachyptera* includes great diversity of floral morphology that is associated with different pollination syndromes. *Pachyptera aromatica* has white, hypocrateriform corollas and nocturnal anthesis (Barbosa Rodrigues 1891). The flowers of this species are classified as “*Tanaecium* type” and fit the hawkmoth pollination syndrome (Gentry 1974). *Tanaecium* type flowers evolved multiple times within Bignonieae (Alcantara and Lohmann 2010) and are found in *Tanaecium* and *Bignonia nocturna* (Barb. Rodr.) L.G. Lohmann. *Pachyptera erythraea*, on the other hand, have orange to red flowers that are tubular campanulate, with sub-exserted anthers. These flowers are classified as “*Martinella* type” and are likely pollinated by hummingbirds (Gentry 1974).

*Pachyptera incarnata*, *P. kerere* and *P. linearis* share infundibuliform and dorso-ventrally compressed corollas, with two longitudinal ridges in the throat that form yellow nectar guides, as well as included anthers. *Pachyptera incarnata* has light pink to pale purple flowers, while *P. kerere* and *P. linearis* have white to cream flowers. These flowers are classified as “*Anemopaegma* type” and are pollinated by large to medium-sized bees, mainly euglossine and anthophorids (Gentry 1974).

### Etymology

*Pachyptera* is a Latin derived name that means “with thick wings” (from Latin: pach = thick, aptera = without wings). This characteristic is found in the type species of the genus, *Pachyptera kerere*.

### Morphology

*Habit.* All species of *Pachyptera* are lianas, although seedlings are initially herbaceous and free standing until ca. 80 cm (grow vertically).

*Stems.* The stems of *Pachyptera* exhibit four phloem wedges in cross-section, a type of cambial variation also found in *Adenocalymma*, *Martinella*, *Cuspidaria*, *Fridericia* and *Tanaecium* (Lohmann 2006, Lohmann and Taylor 2014). Moreover, the pith of the stem of *Pachyptera* is solid although a few specimens of *P. aromatica* also show a slightly hollow pith, a condition only known from *Stizophyllum* and *Pleonotoma* within tribe Bignonieae (Lohmann and Taylor 2014). Cylindrical to tetragonal stems are found in *Pachyptera*, sometimes within a single individual. Young stems are usually cylindrical, becoming tetragonal in more advanced stages of development. Tetragonal stems are only found in stems ≥ 6 cm^2^ of *P. aromatica* but are also found in stems with a smaller diameter in other species of the genus (Francisco personal observation). Stem surface is striated and frequently bears lenticels (except in *P. aromatica*). The peeling bark is a morphological synapomorphy of the genus (Lohmann 2006, Lohmann and Taylor 2014).

**Figure 2.**
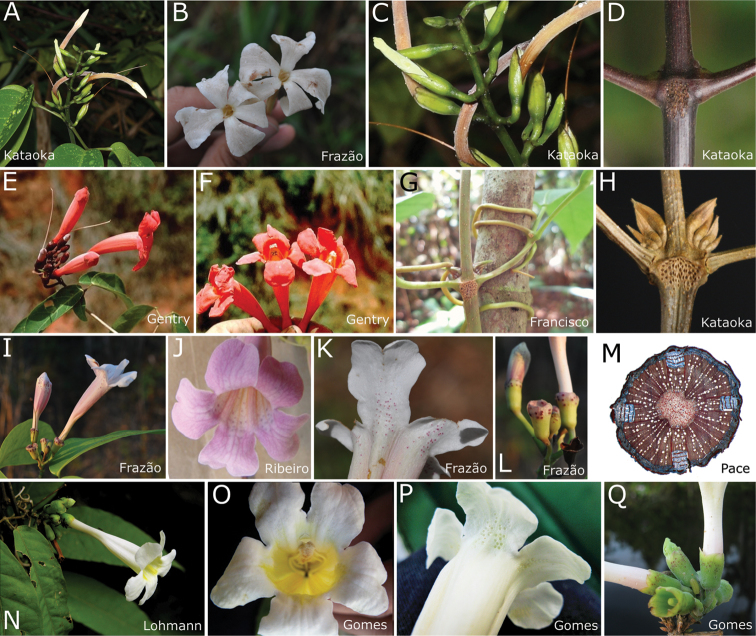
Sample of morphological features of *Pachyptera*. **A–D** *Pachyptera aromatica*: **A** Inflorescence **B** Frontal view of flowers **C** Detail of inflorescence and flowers, showing calyx partition **D** Interpetiolar region of stem with extra floral nectaries (EFNs) and prophylls of the axillary buds 3-seriated, triangular and minute **E–F** *P. erythraea*: **E** Inflorescence **F** Frontal view of flowers **G–L** *P. incarnata*: **G** Stem with tendril surrounding a tree **H** Interpetiolar region of stem with EFNs and prophylls of the axillary buds flattened, ensiform and seriated **I** Inflorescence **J** Frontal view of flower **K** Pink patelliform glands on flower lobes **L** Detail of calyx **M–Q** *P. kerere*: **M** Stem cross-section with four phloem wedges **N** Inflorescence **O** Frontal view of flower **P** White patelliform glands on flower lobes **Q** Detail of calyx.

*Prophylls of the axillary buds.* Prophylls of the axillary buds, referred to as “pseudostipules” in the past (e.g. Gentry 1980), exhibit several shapes and are useful generic characters within Bignonieae (Lohmann and Taylor 2014). Species of *Pachyptera* usually have multiple flattened and ensiform prophylls of the axillary buds (triangular and minute in *P. aromatica*, Fig. 5B), arranged in 3(–5) series (Fig. 2H). Sometimes the prophylls are so minute in *P. aromatica* that only the larger prophyll series is visible to the naked eye (Fig. 2D). The supra-numerary prophylls are an exclusive morphological synapomorphy of *Pachyptera* (Fig. 2G, H).

*Extrafloral nectaries.* Extrafloral nectaries (i.e. EFNs) are useful generic and species level markers within Bignonieae, aiding the identification of sterile materials (Seibert 1948, Lohmann and Taylor 2014). EFNs produce sugar that attracts ants which, in turn, have an important protective role against herbivores (Gentry 1974, Nogueira et al. 2015). In *Pachyptera*, EFNs are composed of large groups of patelliform glands located between the interpetiolar region (Fig. 2D, G, H) and at the petiole apex, right below the junction with the petiolules. Interpetiolar gland fields are also found in other Bignonieae genera (e.g. *Fridericia*, *Lundia*, *Tanaecium*) and have evolved multiple times within the tribe (Nogueira et al. 2013, Lohmann and Taylor 2014). On the other hand, clusters of patelliform glands located on petioles and petiolules are rare in Bignonieae and only known from a few species (e.g. *Tanaecium pyramidatum* and *Mansoa standleyi*).

*Leaves and tendrils.* As with most representatives of Bignonieae, leaves of *Pachyptera* are 2-3-foliate, with the terminal leaflet replaced by a trifid tendril. Tendrils are often deciduous, leaving a tiny scar in the position of tendril detachment. Leaflets can be quite variable in shape, varying even within a single species. Leaflet asymmetry is striking in the group and can help in its identification.

*Inflorescences.* The inflorescence of *Pachyptera* is a simple raceme that can originate from the apical and axillary buds, producing terminal and axillary inflorescences respectively. Racemes can be lax, with a well-developed central axis, ca. 6-24 cm in *P. aromatica* (Fig. 2A, C) or reduced, with a short central axis (< 4.8 cm long) in all other species of the genus (Fig. 2E, I, N). In *Pachyptera*, inflorescence bears ca. 6-30 flowers, although only 1-2 flowers open at a time.

**Figure 3.**
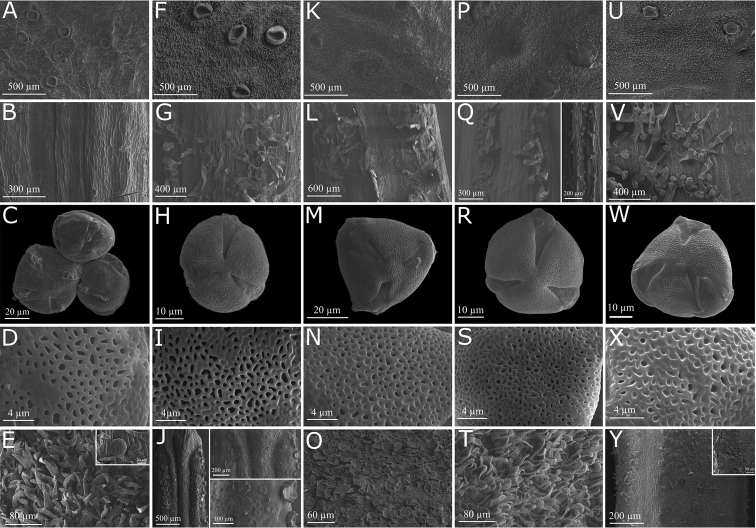
Vertical lines show calyx with patelliform glands, region of stamen insertion, pollen grains, detail of pollen exine and ovary surface variation in all species of *Pachyptera*, respectively. **A–E** *P. aromatica* **F–J** *P. erythraea* **K–O** *P. incarnata* **P–T** *P. kerere* **U–Y** *P. linearis*.

*Calyx.* The calyx of *Pachyptera* is tubular (cupular in *P. erythraea*) with grouped patelliform glands on the upper half (Figs 2M–N, 3A, F, K, P, U), a synapomorphy of the genus. Even though this feature evolved multiple times within the tribe (Lohmann 2006; Lohmann and Taylor 2014), each evolutionary event led to a different gland type and arrangement and the calyx glands found in *Pachytpera* have a unique morphology and arrangement within the tribe. These patelliform glands are conspicuous, sometimes wine-coloured (Fig. 1M) and thought to play an important role against nectar robbers (Gentry 1974).

*Corollas.* More than half of *Pachyptera* species have infundibuliform and dorso-ventrally compressed corollas (Fig. 2I, N), with internal yellow nectar guides (Fig. 2F, O) and a villous portion where stamens and staminodes are included (Fig. 3G, L, Q, V). The corolla tube shape of *P. erythraea* is slightly modified and expands above the short basal constriction and becomes tubular-campanulate (Figs 2E–F, 8B). On the other hand, *P. aromatica* exhibits an extraordinary and distinctive morphology, showing a corolla hypocrateriform, not compressed (Fig. 2A–B), without nectar guides that are glabrous internally (Fig. 3B). Corolla colour is useful for species identification, ranging from white to red (see Figs 1, 2). All species display nectaries on the upper portion of the corolla tube and base of the corolla lobes that exude large globules of colourless and viscous liquid (Fig. 2K, P), likely associated with ant-plant interactions. This feature is a morphological synapomorphy of the genus (Lohmann 2006; Lohmann and Taylor 2014).

*Androecium.* As with most members of Bignonieae, *Pachyptera* has four didynamous stamens and one staminode. Filaments are usually glabrous but puberulous in *P. erythraea*. The anthers are generally included, but sub-exserted in *P. erythraea* (Fig. 2F). The densely villous anthers, with curved thecae, are diagnostic of the majority of *Pachyptera* species (Fig. 2F, O). Only *P. aromatica* has glabrous anthers with straight thecae. Villous anthers are an important feature of *Pachyptera* (except *P. aromatica*), only shared with *Lundia*.

*Pollen.* Pollen has been shown to represent a useful trait for generic delimitation within the Bignoniaceae (Gentry and Tomb 1979). Members of *Pachyptera* generally have microreticulate 3-colpate pollen (Fig. 3H, M, R, W), a condition also found in *Lundia*, *Pleonotoma* and *Tanaecium* (Gentry and Tomb 1979). However, *P. aromatica* has psilate-foveolate to microreticulate 4-colpate pollen grains (Fig. 3C–D).

*Gynoecium.* Members of *Pachyptera* have capitate, elliptic and ovate stigmas. While the style and stigma are always glabrous, the ovary is pubescent (Fig. 3E, T, Y) or lepidote (Fig. 3J, O). As with most representatives of Bignoniaceae, *Pachyptera* has bilocular ovaries, with two ovules per locule and axillary placentation. All species have well-developed nectar discs.

**Figure 4.**
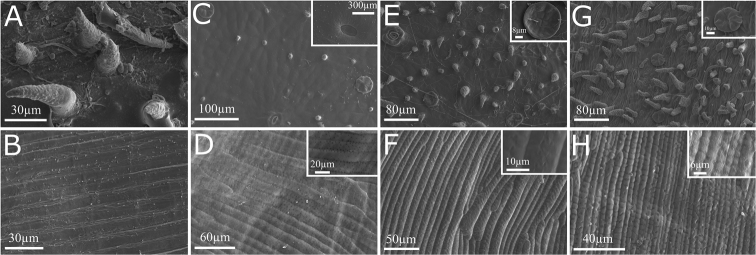
Vertical lines show fruit and seed surface, respectively. **A–B** *P. aromatica* **C–D** *P. incarnata* **E–F** *P. kerere* **G–H** *P. linearis*.

*Fruits.* Fruits are coriaceous to woody septicidal capsules, with two valves. The capsule is linear and flattened in most species (fusiform and inflated in *P. kerere*), puberulous, lepidote, covered with patelliform glandular trichomes and without lenticels (Fig. 4A, C, E, G). Each valve has an inconspicuous longitudinal midline (conspicuous and raised in *P. kerere*).

*Seeds.* The seeds of *Pachyptera* are mostly oblong, thin, chartaceous to coriaceous, with membranaceous and hyaline wings, except from *P. kerere* in which seeds are irregularly circular and obcordate, thick, corky and wingless. Seed surface has provided excellent information for the systematics of various plant groups (Barthlott 1981). The seed surface of *Pachyptera* species is striated, with a distinctive secondary sculpture in each species. More specifically, the seed surface of *P. aromatica* is striated and smooth, while the seed surface of *P. incarnata* is striated with randomly distributed micropores and the seed surface of *P. kerere* is striated with two pairs of medium micropores on each striation. In *P. linearis*, the seed surface is striated, with the striations being regularly interrupted by lateral rays (Fig. 4B, D, F, H). The seed surface of *P. erythraea* is unknown.

## Material and methods

*Species delimitation.* Molecular phylogenetic data (Francisco and Lohmann submitted) was used to aid species delimitation. While it is understood that not all species need to be monophyletic, species are evolutionary lineages that reach a status of reciprocal monophyly in advanced stages of the speciation process (de Queiroz 2007; Funk and Olmland 2003). As such, independent evolutionary units that share a unique combination of features are treated here as separate species, following Cracraft (1983).

*Morphological descriptions.* Morphological descriptions of all species of *Pachyptera* were based on extensive fieldwork and on the analysis of multiple herbarium specimens. The authors examined 378 specimens deposited in the following herbaria: A, B, COL, ESA, F, G, HB, HERBAM, HRCB, HUA, IAN, INPA, K, LINN, MBM, MG, MICH, MO, NY, NX, P, R, RB, RBR, S, SPF, SP SPSF, UEC, UFACPZ, US, VEN and WU (acronyms following Thiers 2015). Images or the actual specimens of all type materials were also analysed. Fieldwork was conducted between 2014 and 2015, in the Brazilian states of Amazonas, Pará and Roraima, the centre of diversity of *Pachyptera*. Specimens collected during field expeditions were deposited at SPF and MO. All accepted names are listed alphabetically, with nomenclatural discussions and citations following McNeill et al. (2012).

Morphological descriptions and measurements were conducted on dried specimens and fresh materials following the terminology of Lohmann and Taylor (2014), with additional terms from Radford (1974), Gentry and Tomb (1979), Hesse et al. (2009), Hickey (1979), Nogueira et al. (2013) and Weberling (1992). Rare conditions are shown within parentheses. Calyx, corolla and ovary surfaces, fruit coat, pollen surface and seed coat were analysed from representative specimens of each taxon using scanning electron microscopy (SEM) (Appendix 1). The selected structures were mounted on stubs and sputter-coated with gold. Micrographs were obtained on a Zeiss DSM 970 scanning electron microscope.

*Distribution maps and examined specimens.* Distribution maps were prepared using QGIS 2.16.3 (QGIS Development Team 2016). A list of examined specimens was prepared and listed alphabetically using the R package monographaR (Reginato 2016) implemented in R (R Development Core Team 2017).

## Taxonomic treatment

### Pachyptera

Taxon classificationPlantaeLamialesBignoniaceae

DC., Prodr. 9: 175. 1845

Pachyptera DC., Prodr. 9: 175. 1845. Type: Pachyptera foveolata DC. (lectotype, designated by Sandwith [1932: 84]) [= Pachyptera kerere (Aubl.) Sandwith]
Sererea Raf., Sylva Tellur. 107. 1838. Type: Sererea heterophylla (Willd.) Raf., Sylva Tellur. 107. 1838. *nom. illeg. superfl.* [= Pachyptera kerere (Aubl.) Sandwith]
Leucocalantha Barb. Rodr., Vellosia, ed. 2. 1: 46, tab. 7. 1891. Type: Leucocalantha aromatica Barb. Rodr., Vellosia. ed. 2. 1: 47, tab. 7. 1891.

#### Description.

*Liana*; stems with four phloem wedges in cross-section, solid (hollow in some specimens of *P. aromatica*), cylindrical to tetragonal, striated, with lenticels (without in *P. aromatica*), with interpetiolar extrafloral nectaries, with a continuous (discontinuous) and transversal interpetiolar ridge, with a papery peeling bark, lepidote, puberulous becoming glabrescent with age; prophylls of the axillary buds 3(–5) seriated (a single series visible to the naked eye in some specimens of *P. aromatica*), flattened and ensiform (triangular and minute in *P. aromatica*). *Leaves* 3-2-foliolate with the terminal leaflet replaced by a trifid tendril; blades discolorous (concolor), chartaceous to coriaceous, usually asymmetric (symetric in some specimens of *P. aromatica*), apex mucronulate, glabrous to puberulous, with simple trichomes covering veins (throughout surface), lepidote, with patelliform trichomes throughout the lamina, venation pinnate, secondary venation brochidromous, tertiary venation percurrent, margin entire, flat or sub-revolute; petioles striated, apices articulated, glabrous to puberulous, lepidote, patelliform glands distributed at petiole apices; petiolules with unequal lengths, striated, apices not-pulvinated (pulvinated in *P. aromatica*), puberulous, lepidote, lateral petiolules shorter than the apical ones. *Inflorescence* axillary or terminal, a few-flowered raceme, congested (lax in *P. aromatica*); axis puberulous, lepidote, patelliform glands grouped at the axis; pedicel puberulous, lepidote; bracts and bracteoles caducous, scarcely evident, puberulous, lepidote. *Calyx* tubular (cupular in *P. erythraea*), coriaceous, smooth, glabrous internally, puberulous externally, lepidote, patelliform glands grouped at the upper portion. *Corolla* white to cream (orange to red in *P. erythraea* and light pink to pale purple in *P. incarnata*), with yellow nectar guides, infundibuliform, (hypocrateriform in *P. aromatica* and tubular-campanulate in *P. erythraea*), straight, dorso-ventrally compressed (not compressed in *P. aromatica*), membranaceous, tube puberulous externally, lepidote, glabrous internally, but villous at the region of insertion of stamens and staminode (glabrous in *P. aromatica*); lobes imbricate, with a pair of patelliform glands arranged in lines externally, lepidote internally. *Androecium* didynamous, included in two heights, with one staminode, glabrous (puberulous in *P. erythraea*); anthers white, becoming darkish with age, included (sub-exserted in *P. erythraea*), villous (glabrous in *P. aromatica*), basifixed, connective thick, round (acute in *P. aromatica*), with thecae divergent, curved forward (straight in *P. aromatica*); pollen 3 colpate, microreticulate (4 colpate, psilate-foveolate-microreticulate in *P. aromatica*). *Gynoecium* glabrous; ovary cylindrical, not-sulcate (bisulcate in *P. erythraea*), smooth, pubescent, (densely lepidote in *P. erythraea* and *P. incarnata*); ovules arranged in two series per locule, placentation axial; stigma glabrous; nectar disc well developed, glabrous. *Capsule* linear, flattened (fusiform, inflated in *P. kerere*), coriaceous to woody, smooth, puberulous, lepidote, with patelliform glandular trichomes throughout, in higher densities at the margins of valves, without lenticels, each valve with an inconspicuous longitudinal midline (conspicuous and raised in *P. kerere*), calyx caducous; seeds oblong, thin, not-corky (irregulary circular, obcordate, thick and corky in *P. kerere*), chartaceous to coriaceous, glabrous, smooth, striated, winged (wingless in *P. kerere*), with membranaceous (chartaceous) and hyaline wings.

#### Nomenclatural note.

*Sererea* Raf. was described to accommodate one species, *Sererea heterophyla* Raf., using a wrong spelling. However, *Sererea heterophylla* (Willd.) Raf. was actually based on *Bignonia heterophylla* Willd, a superfluous name for *Bignonia kerere* Aubl.

#### Number of species, distribution and habitat.

*Pachyptera* comprises five species found in wet and flooded forest vegetation from Belize to Bolívia and Brazil.

#### Key to species of *Pachyptera*

**Table:** 

1	Stems cylindrical (if tetragonal, then only on older portions), without lenticels; prophylls of the axillary buds triangular and minute; corolla hypocrateriform	***P. aromatica***
–	Stems tetragonal (if cylindrical, then only on younger portions), with lenticels; prophylls of the axillary buds ensiform and flattened; corolla infundibuliform or tubular-campanulate	**2**
2	Corolla light pink to pale purple or orange to red; ovary densely lepidote	**3**
–	Corolla white to cream; ovary pubescent	**4**
3	Calyx reddish-wine throughout, cupular; corolla orange to red, tubular campanulate; stamens sub-exserted; capsule linear, 34.0–41.0 cm long, ≥ 2.7 cm wide	***P. erythraea***
–	Calyx green, light pink at the apex, tubular; corolla light pink to pale purple, infundibuliform; stamens included; capsule linear, 10.5–42.6 cm long, ≤ 2.6 cm wide	***P. incarnata***
4	Ovary densely pubescent; capsule fusiform, inflated, each valve with a conspicuous midline; seeds thick, corky and wingless	***P. kerere***
–	Ovary sparsely to moderately pubescent; capsule linear, flat, each valve with an inconspicuous midline; seeds thin, coriaceous and winged	***P. linearis***

### 1. Pachyptera aromatica

Taxon classificationPlantaeLamialesBignoniaceae

(Barb. Rodr.) L.G. Lohmann, Ann. Missouri Bot. Gard. 99(3): 456. 2014

Fig. 5

Pachyptera aromatica (Barb. Rodr.) L.G. Lohmann, Ann. Missouri Bot. Gard. 99(3): 456. 2014. Leucocalantha aromatica Barb. Rodr., Vellosia. ed. 2. 1: 47, tab. 7. 1891. Pachyptera aromatica (Barb. Rodr.) L.G. Lohmann, Cat. Pl. Fung. Brasil 1: 770. 2010, *nom. nud.* Type: Brazil. Amazonas: in capoeiras prope Manáos, in Rio Negro, July, fl., B. Rodrigues 633. Lectotype (designated here): tab. 7, in Vellosia. 1891, excluding the pollen image.

#### Description.

*Liana*; stems solid (hollow), cylindrical (tetragonal when ≥ 6 cm^2^ diameter), vinaceous, with greyish striations, without lenticels; prophylls of axillary buds 3-seriated (a single series visible to the naked eye in some specimens), triangular and minute. *Leaves* with blades discolorous, chartaceous to coriaceous, elliptic, obovate or ovate-lanceolate, asymmetric (symmetric), apex acuminate or caudate, base cuneate, obtuse or rounded, lateral blades 5.3–19.8 × 2.0–8.0 cm, apical blades 9.0–19 × 3.8–9.0 cm; petioles cylindrical, (0.3-)1.0–6.0 cm long; petiolules often pulvinated, lateral petiolules 0.5–6.0 cm long, apical petiolules 3.0–8.5 cm long. *Inflorescence* a lax raceme, 6–24 cm long; pedicel 0.5–1.8 cm long; bracts 0.4–2.1 mm long; bracteoles cymbiform or lanceolate, 0.7–0.8 mm. *Calyx* green, tubular, sub-bilabiate, 5-denticulate (truncate), 1.0–1.8 × 0.3–0.6 cm. *Corolla* white, hypocrateriform, 6.3–12.2 cm long, 0.4–1.0 cm of diameter at the tube mouth; lobes elliptic or obovate, 1.9–4.0 × 1.1–1.9 cm. *Androecium* with the longer stamens 14.0–23.1 mm long, the shorter stamens 11.0–19.7 mm long, glabrous; anthers glabrous, included, with thecae straight, 3.8–6.2 × 0.3–0.6 mm; pollen 4-colpate, psilate-foveolate-microreticulate. *Gynoecium* ca. 5.4 cm long; ovary 2.7–4.8 × 1.0–1.2 mm, cylindrical, not-sulcate, smooth, moderately to densely pubescent, with simple and dentritic trichomes, sparsely lepidote, with glandular peltate trichomes, without patelliform glandular trichomes; stigma ovate, 2.3× 1.6 mm; nectar disc 0.3–1.4 × 1.3–2.1 mm. *Capsule* linear, flattened, 33.1–95.0 × 1.0–2.0 cm, each valve with an inconspicuous longitudinal midline; seeds oblong, 4.0–5.5 × 1.0–1.6 cm, thin, not-corky, chartaceous, striated, secondary sculpture smooth, winged, with membranaceous (chartaceous) and hyaline wings.

**Figure 5.**
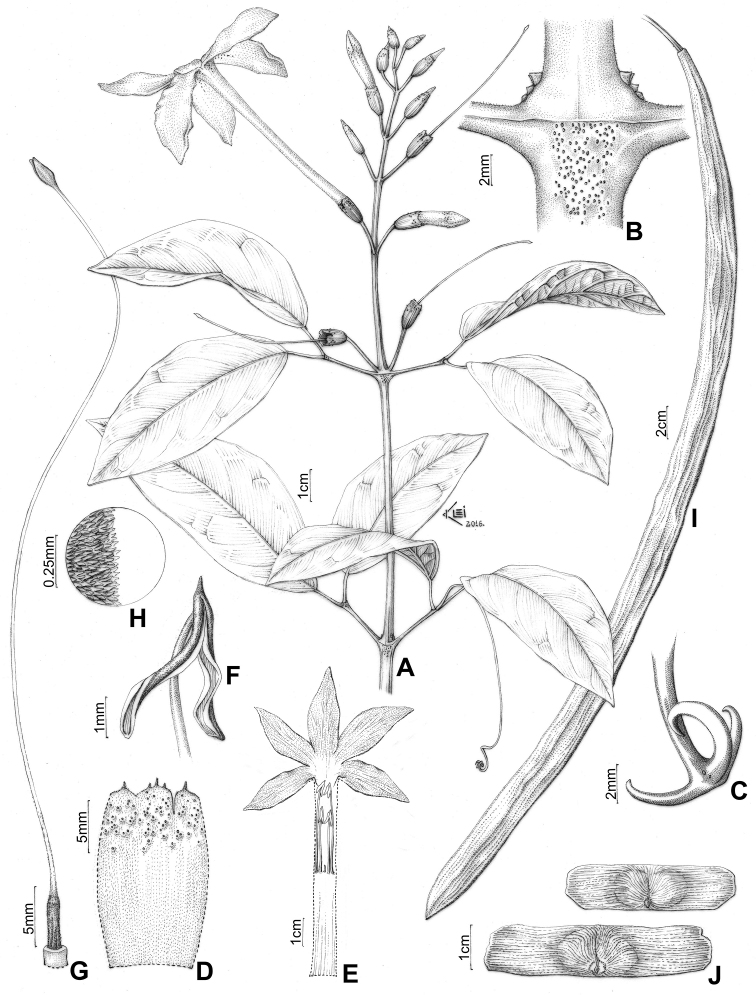
*Pachyptera aromatica*: **A** Flowering branch **B** Interpetiolar region with extra-floral nectaries (EFNs) and prophylls of the axillary buds 3-seriated, triangular and minute **C** Trifid tentril **D** Open calyx (external view) **E** Open flower showing the androecium **F** Upper portion of stamen showing glabrous filament, glabrous anther and acute connective **G** Gynoecium **H** Detail of ovary surface showing pubescent indument (L.H. Fonseca 327, SPF) **I** Fruit linear and flattened **J** Seeds wings (T.B. Croat 11085, MO).

#### Distribution.

**Figure 6.**
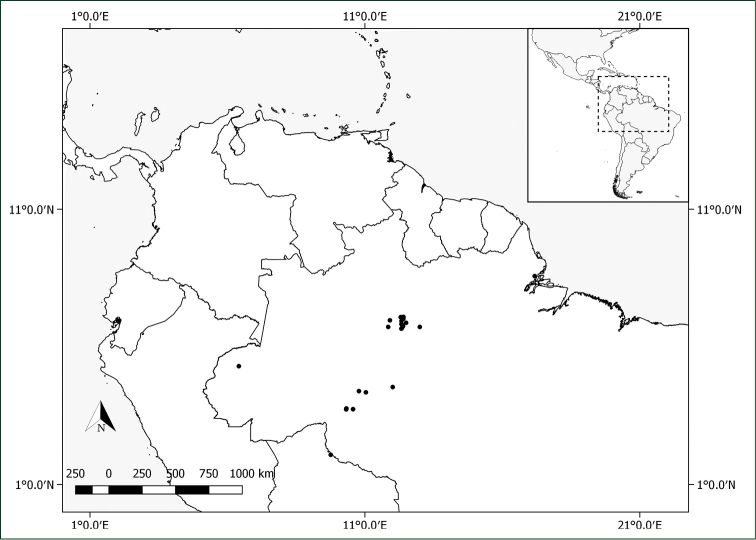
Distribution of *Pachyptera aromatica*.

*Pachyptera aromatica* grows in wet forest vegetation in the Brazilian Amazon (Amapá, Amazonas, Rondônia). Fig. 6.

#### Phenology.

This species flowers from June to January. Fruits were collected in January, March and July through November.

#### Nomenclatural note.

Like Lohmann and Taylor (2014), it was not possible to locate the holotype of *P. aromatica* during multiple visits to the RB, where the holotype was thought to be deposited. It was also not possible to locate any isotypes of any of the collections visited, indicating that the types of *P. aromatica* were likely lost, just like several other type materials collected by Barbora Rodrigues in the Amazon (Mori and Ferreira 1987). As such, we here designate the illustration used in the original description of this species as the lectotype.

#### Taxonomic comments.

*P. aromatica* is characterised by cylindrical (tetragonal when ≥ 6 cm^2^ diameter) and vinaceous stems with greyish striations, interpetiolar extrafloral nectaries, triangular and minute prophylls of the axillary buds 3-seriated and white and hypocrateriform corollas (Figs 2A–D, 5A). This species was originally described within a monotypic genus due to its unusual morphology (Barbosa Rodrigues 1891), but was later transferred into *Pachyptera* based on a combination of morphological and molecular phylogenetic data (Lohmann and Taylor 2014). *Pachyptera aromatica* is sister to the remaining species of the genus (Fig. 1; Francisco and Lohmann 2017, Francisco and Lohmann submitted). The phylogenetic placement of *P. aromatica* corroborates its placement within *Pachyptera* and helps to explain the unusal morphology of this taxon. Selection exerted by differ pollinators may help to explain the floral differences amongst *P. aromatica* and its close relatives. *Pachyptera aromatica* also has a series of other unusual features in the genus such as the poorly developed, triangular and minute prophylls of the axillary buds (vs. well developed, flattened and ensiform prophylls of all other species of *Pachyptera*), glabrous region of stamen and staminode insertion (vs. villous region of stamen and staminode insertion of all other species of *Pachyptera*), glabrous anthers with straight thecae (vs. villous anthers with curved thecae of all other species of *Pachyptera*) and white hypocrateriform corollas (vs. white to red infundibuliform or tubular-campanulate corollas of all other species of *Pachyptera*) (Fig. 5A–B, E–F).

#### Specimens examined.

**BRAZIL. Amapá**: Macapá, Margem de campo, 31 Oct 1980, fl., B. Rabele 1003 (MG). **Amazonas**: Humaitá, near river Livramento, 6 Oct 1934, fr., B.A. Krukoff 6845 (NY, K); Humaitá, Basin of Rio Madeira, on Rio Livramento, 1 Jan 1982, fl., B.A. Krukoff 12511 (INPA); Iranduba, Estrada entre Novo Airão e Manacapurú, 2°54'46.6"S, 60°57'58.8"W, fl., L.H. Fonseca 327 (SPF); Itacoatiara, Rio Solimões, West of Itacoatiara, brazilnut plantattion, EPILOC360, 3°00'S 58°45'W, 100 m, 15 Jan 1990, fr., A.H. Gentry 69107 (MO); Manaus, Mar 1907, fr., M. Labroy 1906 (P); *Ibid.*, 30 July 1929, fl., A. Sucre s.n. (R, RB); *Ibid.*, 30 July 1929, fl., fr., A. Ducke s.n. (MO); *Ibid.*, 31 July 1929, fl., A. Ducke 22698a (P, R); *Ibid.*, 8 Aug 1931, fr., A. Ducke s.n. (R); *Ibid.*, 8 Nov 1931, fr., A. Ducke 22698b (P); Manaus, BR-17, Km 3, 26 Aug 1955, fl., L.F. Coelho INPA1731 (INPA); *Ibid.*, BR-17, km 3, 30 Aug 1955, fl., F.C. Mello s.n. (INPA); *Ibid.*, ca. 80 km N de Manaus, Distrito Agropecuário da SUFRAMA, Rodovia BR 174, km 64, despois 21 km leste na ZF3, Fazenda Porto Alegre, 1 Jan 1962, st., M.H. Nee s.n. (INPA); *Ibid.*, Campos Sales, margem do Igarapé do Buião, 28 Sept 1954, fl., J.C. Almeida INPA137 (INPA); *Ibid.*, Estrada BR-17, 30 Aug 1955, fl., C.M. Francisco s.n. (MG), Luís s.n. (MG); *Ibid.*, Estrada da Forquilha, Margem do igarapé da cachoeira Alta, 22 Aug 1955, fl., J.C. Chagas INPA1701 (INPA); *Ibid.*, Estrada do Aleixo, km 11 past INPA, 2 Dec 1974, fl., A.H. Gentry 13022 (INPA, MO); *Ibid.*, Estrada do igarapé do Tabatinga, 17 Sept 1963, fl., W.A. Rodrigues 5476 (INPA); *Ibid.*, Estrada do Passarinho, 6 Aug 1962, fl., W.A. Rodrigues 4578 (INPA, SPF); *Ibid.*, Ground of INPA at Manaus, 5 Apr 1974, st., A.H. Gentry 11201 (MO), A.H. Gentry 11207 (MO); *Ibid.*, Igarapé do Buião, 15 Oct 1962, st., W.A. Rodrigues 4693 (MO, US); *Ibid.*, Igarapé do Franco, 29 Aug 1957, fl., J.C. Almeida INPA5722 (INPA); *Ibid.*, Loco Cachaeira Grande, 4 July 1943, fl., A. Ducke 239 (NY, K); *Ibid.*, Margem da estrada do Paredão, 3 Aug 1955, fl., J.C. Almeida INPA1540 (INPA); *Ibid.*, Margem do Igarapé do Buião, 19 Aug 1955, fl., J.C. Almeida INPA1687 (INPA); *Ibid.*, Mauazinho, Industrial development, 2°18'57.6"S, 60°04'58.8"W, 50-60 m, 4 Aug 1987, fl., S. Tsugaru B-690 (NY); *Ibid.*, Outskirts of Manaus, road to INPA, boat landing, behind airport, 26 Nov 1974, fr., A.H. Gentry 12862 (INPA, MO, R); *Ibid.*, s.d. fl., Ule U. 4217 (HB, MO); *Ibid.*, Sede do Inpa, Aleixo, depostio do Oficina, 10 July 1972, fl., M. Silva 1024 (INPA, MO); *Ibid.*, Wedge of Rio Negro, a few km N of Manaus, 26 Nov 1974, st., A.H. Gentry 12888 (INPA, MO); *Ibid.*, Rio Negro, Aug 1900, fl., U. Ule 5217 (K); *Ibid.*, Rio Preto da Eva, 2-5 km N of Manaus-Itacoatiara Road at km 79 near Río Preto da Eva, 24 Nov 1974, fl., A.H. Gentry 12832 (MO); *Ibid.*, Road toward Río Negro, 10 km N from Manaus on Estrada Aleixo, 21 Nov 1974, st., A.H. Gentry 12778 (MO, R); *Ibid.*, Rodovia BR-174 Manaus-Presidente Figueiredo, sentido norte., 02°47'53.97"S, 60°02'13.12"W, 87 m, 23 Sept 2016, fl., E. Kataoka 349 (SPF); *Ibid.* (BR-174), próximo a entrada da Reserva de Campinarana do INPA., 2°35'23.76"S, 60°02'2.26"W, 88 m, 23 Sept 2016, fr., A. Frazão 313 (SPF); *Ibid.*, Reserva Florestal Adolpho Ducke 02°53'S, 59°58'W, 15 July 1995, fl., M.J.G. Hopkins 1570 (SPF); *Ibid.*, 15 July 1995, fl., M.J.G. Hopkins 1574 (NYBG); *Ibid.*, 2 Sept 1962, st., A.P. Duarte 7048 (RB); *Ibid.*, Estrada da entrada, 15 Feb 1995, st., M.J.G. Hopkins 1543 (INPA, SPF); *Ibid.*, Nova Prainha, SB-20-ZA, Ponto 02, 10 Sept 1976, st., J.A. Souza INPA61048 (INPA); *Ibid.*. área interna da Reserva Ducke, planta na trilha LO2 entre 1050 e 1100 metros, no interior do gride PPBIO, 15 Oct 2012, st., A. Nogueira 190 (SPF); *Ibid.*, Planta fichada: 2966-24, 15 July 1995, fl., L.G. Lohmann 28 (INPA, SPF); *Ibid.*, próxima a sede da reserva, na área de platô, 02°55'49.0"S, 59°58'23.2"W, 100 m, 5 May 2015, st., C.S. Gerolamo 9 (SPF); *Ibid.*, km 26 on Manaus-Itacoatiara road, 2°18'S, 59°54'W, 80 m, 19 Jan 1990, fl., A.H. Gentry 69308 (MO); *Ibid.*, 23 Nov 1974, st., A.H. Gentry 12815 (INPA, MO); *Ibid.*, Km 16, 3 Dec 1974, fl., A.H. Gentry 13056 (MO); *Ibid*, km 55, 24 Oct 1963, fr., E. Oliveira 2790 (IAN); *Ibid.*, km 26, 30 Sept 1976, st., J.A. Souza INPA61920 (INPA); *Ibid.*, 1 Jan 1976, st., J.A. Souza s.n. (INPA); *Ibid.*, 15 July 1976, st., J.A. Souza INPA71829 (INPA); *Ibid.*, 21 July 1976, st., J.A. Souza INPA71832 (INPA); *Ibid.*, 25 May 1976, st., J.A. Souza INPA71839 (INPA); *Ibid.*, 3 Aug 1976, st., J.A. Souza INPA71828 (INPA); *Ibid.*, 6 Aug 1976 , J.A. Souza INPA71827 (INPA); Novo Airão, Estação Ecológica Anavilhanas, 2°32'08.0"S, 60°50‘49.0"W, 9 Oct 2006, fl., L.G. Lohmann 794 (SPF). **Rondônia**: Porto Velho, 11 Sept 1963, fl., B. Maguire 56679 (MO); *Ibid.*, Guaporé, 1 June 1952, fl., G.A. Black 52-14674 (IAN); *Ibid.*, Rio Madeira, Aug 1936, fl., A. Ducke 35624 (MO, RB, US); *Ibid.*, Aug 1936, fl., A. Sucre s.n. (RB); Rio Cuieras, 2 km below mouth of Rio Brancinho, 11 Sept 1973, fl., G.T. Prance 17773 (INPA, MO, NY). *Ibid.*, Sub-base do Projeto RADAM, aeroporto internacional local, 3 Sept 1975, fl., C.D.A. Mota 18 (INPA); *Ibid.*, Sub-base do Projeto RADAM, aeroporto internacional local, 3 Sept 1975, fr., C.D.A. Mota 26 (INPA); s.loc., 1 Jan 1972, fr., B. Maguire s.n. (INPA).

### 2. Pachyptera erythraea

Taxon classificationPlantaeLamialesBignoniaceae

(Dugand) A.H. Gentry, Phytologia 35(3): 186, fig. 2A. 1977

Fig. 7

Pachyptera erythraea (Dugand) A.H. Gentry, Phytologia 35(3): 186, fig. 2A. 1977. Pachyptera kerere var. erythraea Dugand, Caldasia 7(31): 16. 1955. Mansoa erythraea (Dugand) A. Gentry, Ann. Missouri Bot. Gard. 66(4): 782. 1979 [1980]. Type: Colombia. Santander: 10 leguas al S.E. de Barranca Bermeja, a 9 km de la margen izquierda del Río Opón, 200 m., 26 Aug. 1954, fl., R. Romero-Castañeda 4727 (holotype, COL000004375!).

#### Description.

*Liana*; stem solid, tetragonal (cylindrical when young), green or brown, with greyish striations, lenticellate; prophylls of axillary buds 3(-4)-seriated, flattened and ensiform. *Leaves* with blades discolorous, chartaceous, elliptic, ovate-lanceolate, asymmetric, apex caudate, base cordate, oblique, lateral blades 11.0–19.2 × 4.7–8 cm, apical blades 12.0–12.3 × 5.7–6.2 cm; petioles semi-cylindrical, 3.8–6.5 cm long; petiolules not pulvinated, lateral petiolules 1.3–2.4 cm long, apical petiolules 2.5–4.1 cm long. *Inflorescence* a congested raceme, 2.2–4.5 cm long; pedicel 1.8–2.6 cm long; bracts 1.2–1.3 × 0.5–0.9 mm long; bracteoles cymbiform or triangular, 0.4–0.8 mm. *Calyx* reddish-wine, cupular, truncate, minutely 5-lobed, 0.9 × 0.7–0.9 cm. *Corolla* orange to red, tubular-campanulate, 8.5–8.7 cm long, ca. 2.2 cm of diameter at the tube mouth; lobes rounded (sub-circular), 1.8 × 1.4–1.6 cm. *Androecium* with the longer stamens 44.8–49.3 mm long, the shortest stamens 34.6–39.3 mm long, sparsely puberulous at the dorsal portion of the apex; anthers villous, sub-exserted, with thecae curved forward, 2.0–2.6 × 0.6–1.4 mm; pollen 3-colpate, microreticulate. *Gynoecium* ca. 6.6 cm long; ovary 3.1–3.9 ×1.3–1.6, cylindrical, bisulcate, smooth, sparsely puberulous at grooves, with simples trichomes, moderately lepidote, with patelliform glandular trichomes distributed in two vertical lines parallel to the grooves, glandular peltate trichomes forming a vertical line of transition between internal and external grooves and mixing with simple trichomes within the grooves; stigma elliptic, 3.8 × 1.9–2.1 mm; nectar disc ca. 2.4 × 3.8 mm. *Capsule* linear, flattened, 34.0–41.0 × 2.7–3.0 cm, each valve with an inconspicuous longitudinal midline; seeds oblong, 7.5–9.8 × 2.0–2.1 cm, thin, not-corky, chartaceous to woody, striated, secondary sculpture not seen, winged, with membranaceous and hyaline wings.

**Figure 7.**
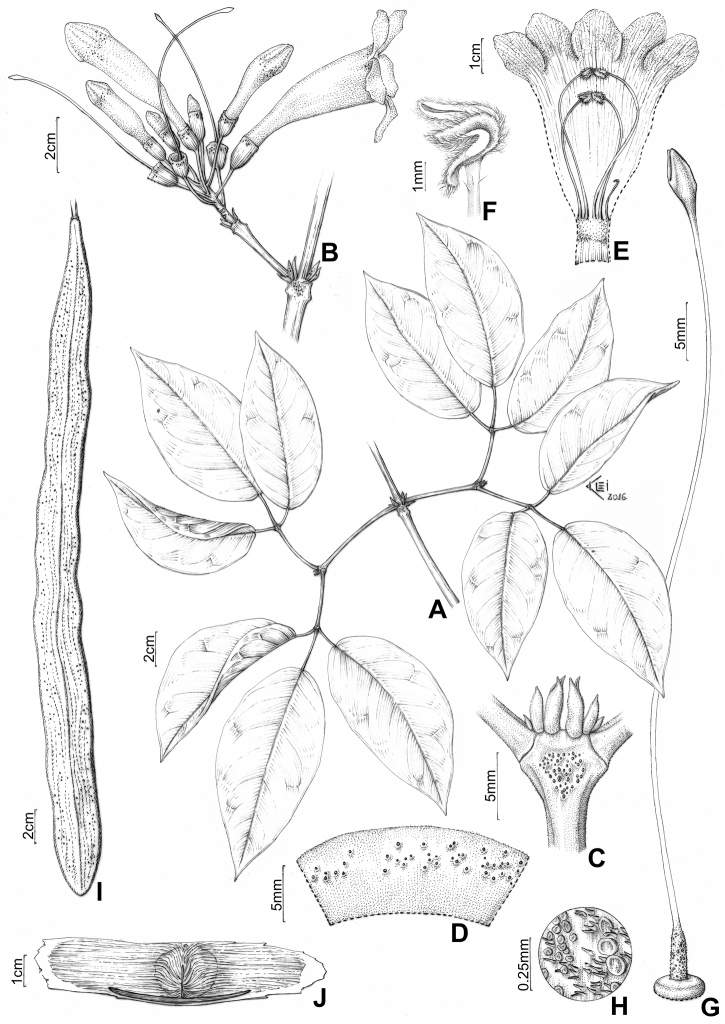
*Pachyptera erythraea*: **A** Branchlets with four leaves **B** Inflorescence **C** Interpetiolar region showing extra floral nectaries and prophylls of axillary buds 3-seriated, flattened and ensiform **D** Open calyx (external view) **E** Open flower showing the androecium with anthers united by the villous indument **F** Upper portion of stamen, showing villous and curved thecae **G** Gynoecium **H** Detail of the lepidote ovary indument, with simples trichomes and glandular peltate, and patelliform trichomes (M. Weir 72, K) **I** Linear and flattened fruit **J** Seed wings (A.H. Gentry 20050, MO).

#### Distribution.

**Figure 8.**
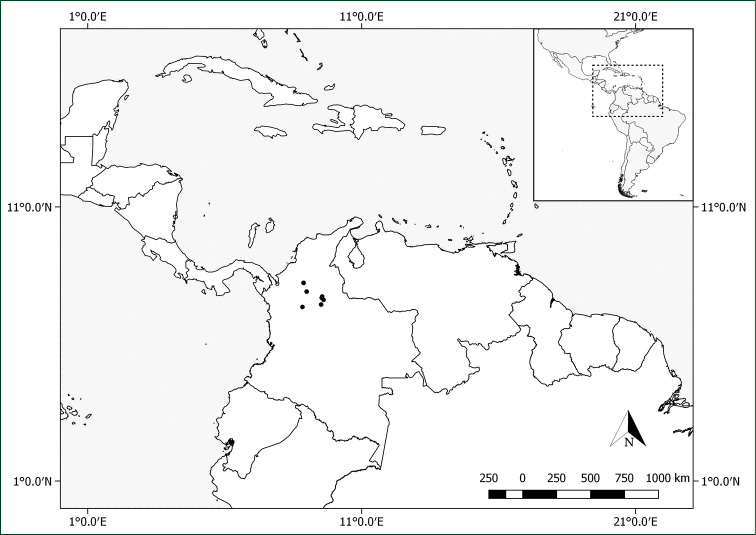
Distribution of *Pachyptera erythraea*.

*Pachyptera erythraea* is endemic to wet forest vegetation from the Magdalena River Valley in northern Colombia (Antioquia, Santander). Fig. 8.

#### Phenology.

Flowers collected in January, March and July to December. Two fruiting collections are known, one collected in July and the other in September.

#### Taxonomic comments.

*Pachyptera erythraea* is distinguished by the orange to red corollas (vs. white, light pink to pale purple in all other species), cupular calyces (vs. tubular in all other species) and sub-exserted anthers (vs. included anthers in all other species). Moreover, *P. erythraea* is the sole species of *Pachyptera* with tubular-campanulate corollas. The fruit of *P. erythraea* is flat, without a visible longitudinal midline. The seeds are thin and winged, similar to those of *P. incarnata* and *P. linearis*. *Pachyptera erythraea* is closely related to *P. incarnata*, with which it shares a moderately to densely lepidote ovary (Fig. 1). However, these species are separated by the bi-sulcate ovary, with glandular peltate and patelliform trichomes arranged in vertical lines in *P. erythraea* vs. the non-sulcate ovary, fully covered by glandular peltate trichomes in *P. incarnata*.

#### Specimens examined.

**COLOMBIA. Antioquia**: Caucasia, along road to Nechi 24 km from Caucasia-Planeta Rica road, Hacienda Costarica, margin of primary forest and trees remaining in cleared pasture, 8°03'36.0"N, 75°04'48.0"W, 60 m, 21 Mar 1987, fl., J.L. Zarucchi 4887 (HUA, K, MO); *Ibid.*, 21 Mar 1987, fl., J.L. Zarucchi 4862A (MO); Zaragoza, Carretera a Zaragoza entre Carralao y Angostura, 70 m, 13 Jan 1989, fl., G. Ramiro Fonnegra 2580 (HUA); Rio Magdalena, July 1868, fl., M. Weir 72 (K). **Santander**: Barranca Bermeja, 10 leguas al SE de Barranca Bermeja, 7°03'36.0"N, 73°51'36.0"W, 200 m, 26 Aug 1954, fl., R. Romero-Castañeda 4727 (COL, MO); *Ibid.*, 12 leguas al SE de Barranca Bermeja, orilla derecha del rio Opón, 200 m, 4 Oct 1954, fl., R. Romero-Castañeda 4979 (COL); *Ibid.*, 2 km S. of Llanitas, 19 km N. of Barranca Bermeja, 7°03'36.0"N, 73°51'36.0"W, 160 m, 24 July 1975, fl., A.H. Gentry 15369 (MO); 11-13 km N of Barranca Bermeja on road to Puerto, 07°09'19"N, 73°50'28"W, 160m, 24 July 1975, fl., A.H. Gentry 15372 (MO); Campo Capote, Magdalena Valley, campo Capote, 30 km E of Carare, 6.61, -73.91, 300 m, 29 Sept 1977, fr., A.H. Gentry 20050 (MO); El Centro, 3 km S. of El Centro on road to Yarima, 200 m, 25 July 1975, fl., fr., A.H. Gentry 15402 (MO).

### 3. Pachyptera incarnata

Taxon classificationPlantaeLamialesBignoniaceae

(Aubl.) Francisco & L.G. Lohmann comb. nov.

urn:lsid:ipni.org:names:77175179-1

Fig. 9

Bignonia incarnata Aubl., Hist. Pl. Guiane. 2: 645, tab. 261, 262, fig. 1–8. 1775. Bignonia incarnata Aublet sec. Splitg., Tijdschr. Nat. Geschied 9: 7. 1842. nom. nud. Cydista incarnata Miers, Proc. Roy. Hort. Soc. London. 3: 192. 1863. *nom. nud.*Pachyptera kerere var. incarnata (Aubl.) A.H. Gentry, Brittonia 25(3): 235. 1973. Mansoa kerere var. incarnata (Aubl.) A.H. Gentry, Ann. Missouri Bot. Gard. 66(4): 783. 1979 [1980]. Type: French Guiana. s.loc., s.d., (fl., fr), J.B.C.F. Aublet s.n. Lectotype (designated here): tab. 261 and 262, in Hist. Pl. Guiane 1775.

#### Description.

*Liana*; stems solid, tetragonal (cylindrical when young), green or brown (reddish), with greyish striations, lenticellate; prophylls of axillary buds 3(–5)-seriated, flattened and ensiform. *Leaves* with blades discolorous (concolor), chartaceous to coriaceous (membranaceous), elliptic, obovate or ovate-lanceolate, asymmetric, apex acute, acuminate or caudate (retuse), base cordate, oblique, lateral blades 2.6–21.1 × 1.9–9.2 cm, apical blades 5.7–20 × 2.6–7.6 cm; petioles semi-cylindrical, 0.8–5.5 cm long, petiolules not puvinated, lateral petiolules 0.8–5.5 cm long, apical petiolules 1.4–4.4 cm long. *Inflorescence* a congested raceme, 0.8–3.5 cm long; pedicel 0.5–1.3(–7.5) cm long; bracts 0.8–2.5 × 0.7–0.9 mm long; bracteoles cymbiform or filiform, 0.2–0.7 mm. *Calyx* green, light pink at apex, tubular, bilabiate, minutely 5-lobed or truncate, 0.4–1 × 0.4–0.6 cm. *Corolla* light pink to pale purple, infundibuliform, 2.9–7.6 cm long, 0.7–1.9 cm of diameter at the tube mouth; lobes rounded (sub-circular), 0.3–1.5 × 0.4–1.3 cm. *Androecium* with the longer stamens 23.4–17.0 mm long, the shorter stamens 10.0–15.7 mm long, glabrous; anthers villous, included, with thecae curved forward, 2.6–3.1 × 0.5–0.5 mm; pollen 3-colpate, microreticulate. *Gynoecium* 3.0–5.0 cm long; ovary 1.8–2.9× 0.7–0.91, cylindrical, not-sulcate, smooth, glabrous, densely lepidote, with glandular peltate trichomes, rarely with some patelliform glandular trichomes; stigma capitate or ovate, 0.8–3.5 × 0.5 mm; nectar disc 0.8– 1.0 × 1.7–1.8 mm. *Capsule* linear, flattened, 10.5–42.6 × 1.4–2.6 cm, each valve with an inconspicuous longitudinal midline; seeds oblong, 4.0–7.0 × 1.3–2.8 cm, thin, not-corky, chartaceous to sub-coriaceous, striated, secondary sculpture with randomly distributed micropores, winged, with membranaceous and hyaline wings.

**Figure 9.**
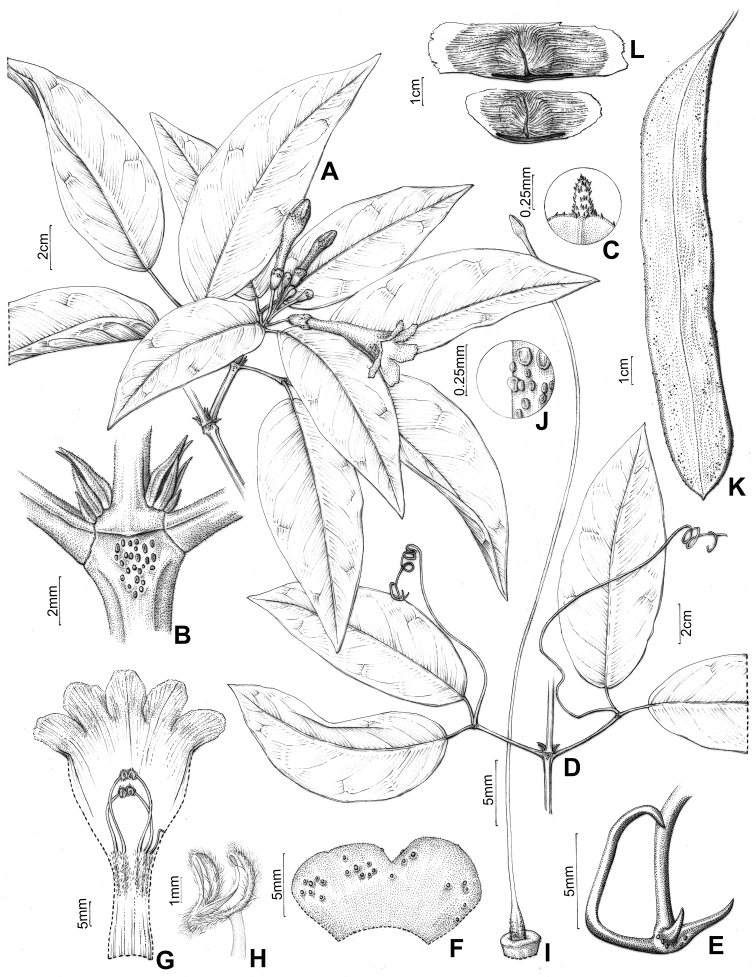
*Pachyptera incarnata*: **A** Flowering branch **B** Interpetiolar region with EFNs and prophylls of axillary buds 3-seriated, flattened and ensiform **C** Mucronulate leaflet apex **D** Branchlets trifoliate with terminal leaflet replaced by trifid tendril **E** Trifid tentril **F** Calyx external view **G** Open flower showing the androecium with anthers united **H** Stamen with villous and curved thecae **I** Gynoecium **J** Ovary surface lepidote, with glandular peltate trichomes (J.N.C. Francisco 103, SPF) **K** Fruit linear flattened capsule **L** Seeds wings (J.N.C. Francisco 122, SPF).

#### Distribution.

**Figure 10.**
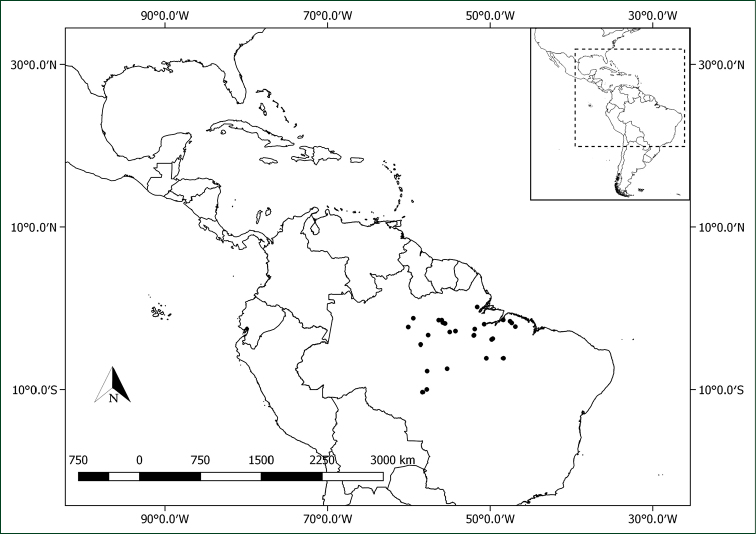
Distribution of *Pachyptera incarnata*.

This species is found in wet forest vegetation in Brazil (Amazonas, Mato Grosso, Pará, Rondônia) and French Guiana. Fig. 10.

#### Phenology.

*Pachyptera incarnata* flowers in February to May and July to December. Fruiting material has been colected in April, May, July to October and December.

#### Etymology.

The specific epithet “*incarnata*” refers to the corolla colour referred by Aublet as “of flesh”.

#### Nomenclatural note.

This species was first described by Aublet (1775) as *Bignonia incarnata*. Gentry (1973) treated *B. incarnata* as a variety of *P. kerere* due to the shared racemose inflorescences, corolla infundibuliform, villous anthers and prophylls of the axillary buds 3-seriated. Gentry (1973) distinguished the two varieties based on differences in the fruit and seed morphology. More specifically, P. kerere var. kerere included the individuals with inflated fruits, corky and wingless seeds, while P. kerere var. incarnata included the individuals with flattened fruits, thin and winged seeds. Despite the floral similarity between these two species, *P. incarnata* is phylogenetically more closely related to *P. erythraea*, with which it shares a densely lepidote ovary and flattened and linear fruits (Fig. 1). Based on the authors’ new molecular phylogeny (Francisco and Lohmann submitted) and morphological data, this taxon is raised back to species-level, following Aublet (1775). As it was not possible to locate original material, the original illustration is here designated as the lectotype.

#### Taxonomic comments.

*Pachyptera incarnata* is characterised by the infundibuliform and light pink to pale purple corolla with ovary densely lepidote. The capsule is linear, flattened and coriaceous, with pink patelliform glandular trichomes throughout the surface and has an inconspicuous longitudinal midline. Seeds are oblong, thin, chartaceous to sub-coriaceous, winged, with membranaceous and hyaline wings.

#### Specimens examined.

**BRAZIL. Amapá**: Campaipi, Embrapa reserve and vicinity, 0°10'N, 51°37'W, 3 Sept 1983, fl., S.A. Mori 15783 (MG, MO). **Amazonas**: Manaus, 31 Aug 1931, fl., A. Ducke 24091 (R); *Ibid.*, estrada do Aleixo, near Manaus, turn off to Río Negro at km 11 past INPA, 2 Dec 1974, fl., A.H. Gentry 13027 (MO); *Ibid.*, INPA boat landing behind Manaus airport, Río Negro, 15 Dec 1974, fr., A.H. Gentry 13323 (MO); *Ibid.*, across from Guarara factory, 20 Apr 1974, fl., D.G. Campbell P22008 (MO); Presidente Figueiredo, Balbina, Rebio Uatumã, grade do PPBio, 6 Oct 2006, fr., J.R. Carvalho-Sobrinho 1078 (INPA). **Mato Grosso**: Aripuanã, MT-420, beira do rio, 10°15'00.0"S, 59°07'12.0"W, 11 July 1997, fl., G.F. Árbocz 4256 (ESA); Juruena, beira do Rio Juruena, floresta aluvial, 10°18'36.0"S, 58°19'48.0"W, 10 July 1997, fl., V.C. Souza 18583 (ESA); Nova Bandeirantes, estrada Iporã, 255 m, 22 July 2015, fl., fr., R.S. Ribeiro 78 (SPF). **Pará**: Belterra, Floresta Nacional do Tapajós, estrada para comunidade de Jamaraguá, km 72, 02°55'15.9"S, 55°01'39.4"W, 114 m, 16 Sept 2015, fl., J.N.C. Francisco 89 (SPF); estrada do Mocambo, IPEAN, 02 May 1969, fl., J.M. Pires 12075 (IAN, MO); Irituia, Rio Irituia, varzea S. Miguel do Guamá, 29 Oct 1948, fl., G.A. Black 48-3355 (IAN); Itaituba, estrada Santarém-Cuiabá, BR 163, km 794, 7°25'S, 55°20'W, 12 May 1983, fl., I.L. Amaral 1248 (MO); Marabá, Marabu, Serra Norte, Carajás, 7°42'36.0"S, 57°48'36.0"W, 01 Aug 1983, fl., M. Silva 1604 (MO, UEC); Óbidos, beira do Lago Curumu, floresta de várzea, 01°51'37.3"S, 55°38'47.3"W, 24 m, 23 Sept 2015, fl., J.N.C. Francisco 130 (SPF); Óbidos, lago Maria Teresa, floresta de várzea, 01°52'37.7"S, 55°35'28.7"W, 14 m, 23 Sept 2015, fl., J.N.C. Francisco 121 (SPF); *Ibid.*, 01°52'38.2"S, 55°35'27.4"W, 14 m, 23 Sept 2015, fr., J.N.C. Francisco 122 (SPF); Oriximiná, Floresta Nacional de Saracá-Taquera, próximo ao alojamento Pioneiros de pesquisadores, floresta de terra firme, 01°27'56.6"S, 56°22'43.8"W, 71 m, 27 Sept 2015, fl., J.N.C. Francisco 151 (SPF); *Ibid.*, Porto Trombetas, rejeitos, Linha 69, beira de floresta, 1°45'36.0"S, 55°51'36.0"W, 09 Dec 1987, fl., O.H. Knowles 1120 (INPA); *Ibid.*, Porto Trombetas, Serra Assas, descampado, 21 Oct 1987, fl., O.H. Knowles 1106 (INPA); Palestina do Pará, fazenda Andorinha sede 2, início da mata do rio Gameleira, 6°06'36.0"S, 48°24'36.0"W, 160 m, 18 Apr 2004, fr., G. Pereira-Silva 8765 (CEN); Parauapebas, Serra dos Carajás, Platô N2, vegetação de canga, 7 Mar 2010, fl., L.C.B. Lobato 3870 (MG); *Ibid.*, à margem da estrada Raymundo Mascarenhas, 8 Feb 1990, fl., J.B.P. Rocha 701 (IAN); Portel, 1°57'36.0"S, 50°45'00.0"W, 21 Oct 1955, fl., L. Williams 18222 (IAN, MO); Porto Trombetas, Mineração Rio do Norte, 1991, fr., Evando 542 (INPA); Santarém, beira da PA-370, próxima à guarita da Usina Hidrelétrica Curuá-Uma, floresta de terra firme, 02°49'21"S, 54°17'58.9"W, 49 m, 19 Sept 2015, fl., J.N.C. Francisco 103 (SPF); *Ibid.*, ramal próximo à Usina Hidrelétrica Curuá-Uma, solo areno argiloso, floresta de terra firme, 02°48'45.2"S, 54°18'08.8"W, 47 m, 19 Sept 2015, fl., J.N.C. Francisco 105 (SPF); São Miguel do Guamá, Rio Guamá, beira do rio, igapó, 21 Aug 1948, fl., fr., Dardano 48-3092 (IAN); Senador José Porfirio, margem direita do Rio Xingu, capoeira de terra firme, 02°34'00"S, 51°55'00"W, 3 Dec 1991, fr., G. Santos 282 (MG); Tucuruí, área de desmatamento, 1 Sept 1983, fl., F.E. Miranda 362 (NY); *Ibid.*, BR-422, Km 45, Breu Branco, margem do rio Tocantins, 5 Nov 1983, fl., J. Ramos 1011 (INPA); *Ibid.*, estrada para o lago 31 de março, 30 Aug 1983, fl., J. Revilla 8397 (NY); *Ibid.*, margens da PA-149 até ca. Km 50, 22 Aug 1983, fr., J. Revilla 8326 (NY); Viseu, Serra do Piriá, à 13km de Açaiteua, 4 Dec 1993, fl., J. Sales 1539 (MG); Vitória do Xingu, 3°19'32"S, 52°00'16"W, 1 Aug 2015, fl., R.V. Pyramo PSACF_EX06147 (RB); V *Ibid.*, 3°22'4"S, 52°02'23"W, 12 Aug 2015, fl., B.R. Silva PSACF_EX06201 (RB).

### 4. Pachyptera kerere

Taxon classificationPlantaeLamialesBignoniaceae

(Aubl.) Sandwith, Recueil Trav. Bot. Néerl. 34: 219. 1937.

Fig. 11

Bignonia kerere Aubl., Hist. Pl. Guiane 2: 644, tab. 260. 1775, excluding the fruit description and tab. 263. Bignonia heterophylla Willdenow, Sp. Pl. 3: 298. 1800 [1801]. nom. superfl. illeg. Sererea heterophyla (Willd.) Rafinesque, Sylva Tellur. 107. 1838. nom. superfl. illeg. Adenocalymma kerere (Aubl.) Bureau & K. Schum. Fl. Bras. 8(2): 119. 1891. Adenocalymma stridula Miers, Ann. Mag. Nat. Hist. ser. 3 7: 392. 1861. *nom. illeg.*, Petastoma kerere (Aubl.) Schnee in. Pittier, Cat. Fl. Venez. 2: 404.1947. Mansoa kerere (Aubl.) A. Gentry, Ann. Missouri Bot. Gard. 66(4): 783. 1979 [1980]. Type: French Guiana. Cayenne, s.d., fl., J.B.C.F. Aublet s.n. (holotype, BM000992379!).
Pachyptera foveolata DC., Prodr. 9: 175. 1845. Adenocalymma foveolatum (DC.) Baillon, Hist. Pl. 10: 7, fig. 9–16. 1891. Adenocalymma foveolatum (DC.) K. Schumann, Nat. Pflanzenfam 4(3b): 214, fig. 89 F–G. 1894. nom. superfl. illeg. Adenocalymma foveolatum (Bureau) Bureau & K. Schumann, Fl. Bras. 8(2): 109. 1896. *nom. illeg.* Type: French Guiana, s.loc., 1819-1821, fr., M. Poiteau s.n. (lectotype, designated by Sprague and Sandwith 1929, p. 84: G-DC [G00014105]!).
Adenocalymma brachybotrys DC., Prod. 9: 202. 184. Type: French Guiana. s.loc., 1821, fl., G.S. Perrottet s.n. (holotype, P03578200!).
Adenocalymma symmetricum Rusby, Descr. S. Amer. Pl. 122. 1920. Type: Venezuela. Lower Orinoco, 1896, fl., Rusby & Squires s.n. (holotype, NY00313053!).
Bignonia benensis Britton ex Rusby, Bull. Torrey Bot. Club 27: 70. 1900. Type: Bolívia. Junction of Beni and Madre de Dios rivers, Aug. 1886, fl, H.H. Rusby 1143. (lectotype, designated here, NY00313133!; isotype, MICH01115822!, NY00313132!, US00603898!, US00125816!).
Tanaecium zetekii Standley, Contr. Arnold Arbor 5:140. 1933. Type: Panamá. Barro Colorado Island, 3 Feb 1932, fl., R.H. Woodworth 363. (holotype, F651874!; isotype, A00093244!, MO807829!, US00125783!).

#### Description.

*Liana*; stems solid, tetragonal (cylindrical in younger portions), green or brown, with greyish striations, lenticellate; prophylls of axillary buds 3(–5)-seriated, flattened and ensiform. *Leaves* with blades discolorous (concolor), membranaceous to chartaceous (coriaceous), elliptic, obovate or ovate-lanceolate, asymmetric, apex acute, acuminate or caudate, base cordate, oblique, lateral blades 4.4–22.5 × 2.1–14.3 cm, apical blades 5.2–22.5 × 2.0–11.5 cm; petiole semi-cylindrical, 0.3–6.9 cm long, petiolules not puvinated, lateral petiolules 0.3–6.0 cm long, apical petiolules 0.8–6.0 cm long. *Inflorescence* a congested raceme, 0.6–4.8 cm long; pedicel (0.2–)0.5–5.7(–7.5) cm long; bracts 1.1–2.4 mm long; bracteoles cymbiform or filiform, 0.4–2.3 × 0.5–0.8 mm. *Calyx* green, sometimes with purplish apex, tubular, bilabiate or sub-bilabiate, truncate, minutely 5-lobed, 0.5–1.2 × 0.4–0.9 cm. *Corolla* white to cream, infundibuliform, 4.0–9.5 cm long, 0.9–2.5 cm of diameter at the tube mouth; lobes rounded (sub-circular), 0.6–1.9 × 0.5–1.8 cm. *Androecium* with the longer stamens 18.0–29.1 mm long, the shorter stamens 11.9–20.3 mm long, glabrous; anthers villous, included, thecae curved forward, 1.9–3.1 × 0.3–1.0 mm; pollen 3-colpate, microreticulate. *Gynoecium* 3.2–6.0 cm long; ovary 1.8–3.6 × 0.8–1.6, cylindrical, not-sulcate, smooth, densely pubescent, with simple and dentritic trichomes, sparsely lepidote, with glandular peltate trichomes, without patelliform glandular trichomes; stigma capitate or ovate, 1.6–3.1 × 0.9–3.5 mm; nectar disc 0.4–1.9 × 0.5–4.0 mm. *Capsule* fusiform, inflated (slightly flattened), 8.0–26.0 × 1.5–3.6 cm, each valve with a conspicuous and raised longitudinal midline; seeds irregularly circular and obcordate, 2.8–4.4 × 1.4–3.0 cm, thick, corky, striated, secondary sculpture with two pairs of medium micropores on each striation, wingless.

**Figure 11.**
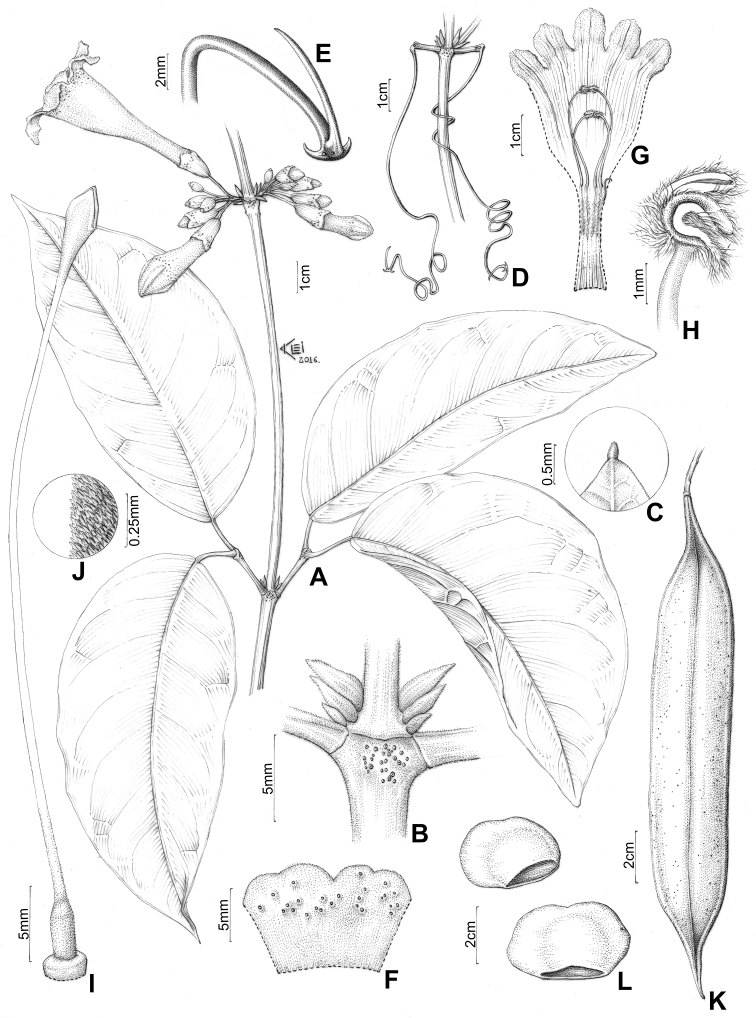
*Pachyptera kerere*: **A** Flowering branch **B** Interpetiolar region with EFNs and prophylls of axillary buds 3-seriated, flattened and ensiform **C** Apice of the leaflet mucronulate **D** Branchlets with terminal leaflet replaced by trifid tendril **E** Trifid tentril **F** Calyx external view **G** Open flower showing the androecium with anthers united **H** Stamen with villous and curved thecae **I** Gynoecium **J** Ovary surface pubescent (J.N.C. Francisco 41, SPF) **K** Fruit fusiform and inflated, with a conspicuous and raised longitudinal midline on valve (T.B. Croat 11085, MO) **L** Seeds corky and wingless (R.A.A. Oldeman B-1449, MO).

#### Distribution.

**Figure 12.**
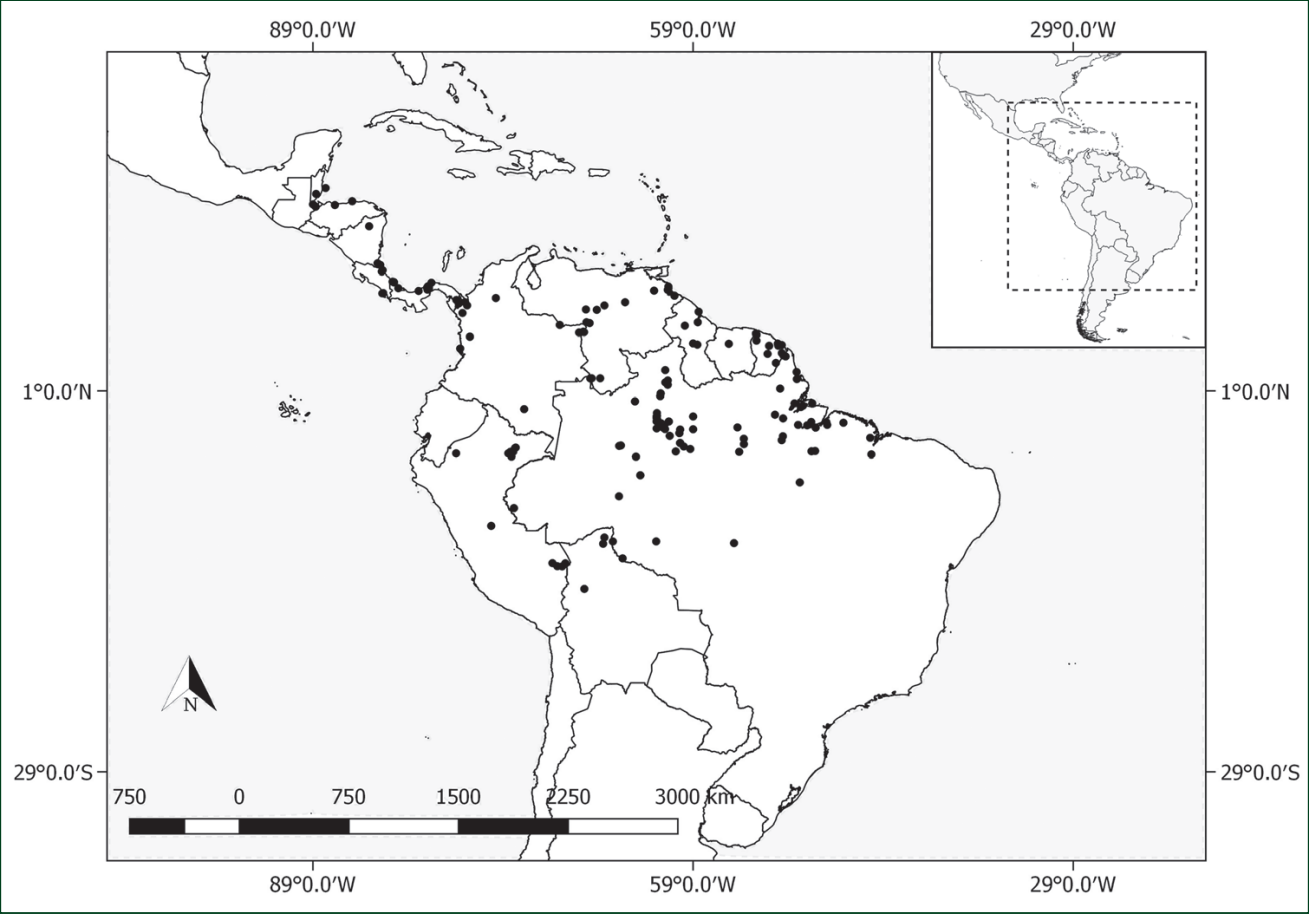
Distribution of *Pachyptera kerere*.

This species is typically found in wet and often flooded forest vegetation in Belize (Toledo), Costa Rica (Limón, Puntarenas), Guatemala (Izabal), Honduras (Atlántida, Cólon), Nicaragua (Atlántico Sur, San Juan), Panama (Bocas del Toro, Colón, Darién, Panama), Bolívia (Beni), Brazil (Acre, Amapá, Amazonas, Maranhão, Mato Grosso, Pará, Rondônia, Roraima), Colombia (Amazonas, Antioquia, Bolívar, Chocó), French Guiana (Cayenne), Guyana (Cuyuni-Mazaruni, Upper Demerara-Berbice, Venezuela), Peru (Amazonas, Huánuco, Loreto, Madre de Dios), Suriname and Venezuela (Amazonas, Apure, Bolívar, Delta Amacuro). Fig. 12.

#### Phenology.

This species flowers and fruits throughout the year.

#### Etymology.

The specific epithet is derived from vernacular name “kéréré” or “téréré” adopted by the indigenous group Galibis, from French Guiana, who use this plant as rope material.

#### Nomenclatural note.

The original description of this species by Aublet (1775) included a mistake in the fruit illustration and description, which consists of the description of *Amphilophium magnollifolium* (Kunth) L.G. Lohmann instead.

*Bignonia benensis* was described by Britton (1990) based on a collection by *Rusby 1143*. This material cannot be considered a holotype because it was not cited in the protologue. Two related specimens are deposited at NY and US and one at MICH. Although there are two materials deposited at NY, only one is original material. The the other specimen is a duplicate that was originally deposited at the College of Pharmacy Herbarium, where Britton worked. Materials from this herbarium were only later incorporated into the NY collection along with other items. Therefore, the specimen originally deposited at NY (NY313133) is here designated as lectotype.

#### Taxonomic comments.

*Pachyptera kerere* is easily recognised by the infundibuliform, white to cream corollas, with densely pubescent ovary. The fusiform, woody, inflated (sometimes slightly flattened) fruit, with a conspicuous and raised longitudinal midline, is very distinctive. The seeds are irregularly circular and obcordate, corky and wingless.

*Pachyptera kerere* shares infundibuliform corollas with *P. incarnata* and *P. linearis*. However, *P. kerere* can be differentiated by the densely pubescent ovary (vs. sparsely pubescent ovary of *P. linearis* and densely lepidote ovary in *P. incarnata*). Furthermore, *P. kerere* is easily separated from *P. incarnata* by the white corolla (vs. light pink to pale purple corolla of *P. incarnata*).

#### Specimens examined.

**BELIZE. Belize**: Mile 5 3/4, Northen Highway, 7 June 1974, fl., J.D. Dwyer 12737 (MO). Stann Creek: 16°50'N, 88°30'W, May 1901, fl., fr., E.J.F Campbell 95 (K). **Toledo**: Maya Mountains foothill, Solomon Camp, vicinity of the junction of Richardson Creek and Bladen Branch, 16°31'48.0"N, 88°45'00.0"W, 80 m, 5 Mar 1987, fl., G. Davidse 32046 (MO). **COSTA RICA. Limón**: Parque Nacional Tortuguero, Estación Agua Fría, alrededores de la casa-estación, vegetación secundaria y relictos de vegetación primaria, 10°24'36"N, 83°33'36"W, 40 m, 24 Oct 1987, fl., R. Robles 1121 (MO); *Ibid.*, Estación Cuatro Esquinas, 800 m al Sur de la casa-estación, a orillas de la Laguna de Tortuguero, 10°30'36.0"N, 83°30'00.0"W, 2 m, 29 Nov 1987, fr., R. Robles 1391 (MO); Puerto Viejo de Talamanca, along road in vicinity of beach between Punta Cocles and Punta Uva, E of Puerto Viejo de Talamanca, 9°37'48"N, 82°42'36"W, 0–5 m, 6 Nov 1984, fl., M.H. Grayum 4411 (MO); Rio Gandoca, Refugio Gandoca-Manzanillo Low-lying coastal swamps and forests, Gandoca (slightly to N of trail from Mata de Limón), 9°36'N, 82°36'W, 0 m, 27 Jan 1987, fl., M.H. Grayum 8032 (MO); Talamanca, Sixaola, Gandoca, finca Cangrejo, Anai, 9°34'45"N, 82°36'20"W, 10 m, 24 Mar 1995, fr., G. Herrera 7551 (K). **Puntarenas**: Cantón de Osa, cuenca Térraba-Sierpe, Chocuaco, 8°43'50"N, 83°27'17"W, 150 m, 29 Dec 1996, fl., R. Aguilar 4824 (MO); Golfo Dulce, Reserva Forestal Golfo Dulce Aguabuena, sector sur, 08°42'00"N, 83°31'12"W, 50 m, 15 Jan 1992, fl., R. Aguilar 818 (MO). **GUATEMALA. Izabal**: Dartmouth, between Dartmouth and Morales toward Lago Izabel, Montana del Mico, 15°30'36.0"N, 88°46'48.0"W, 35 m, 7 Apr 1940, fl., fr., J.A. Steyermark 39022 (F, MO); Puerto Barrios, near Rio Pargueña, 38–40 km N of Puerto Ayacucho, 25 May 1939, fl., P.C. Standley 73082 (F). **HONDURAS. Atlantida**: Esparta, 41.5 km E of Tela on the Tela-Ceiba Hwy then ca. 6 km N along old timber road. In remaining patches of primary forest, 15°39'N, 87°16'W, 100 m, 24 Apr 1994, fl., fr., A.E. Brant 2917 (MO). **Colón**: Rio Guaimoreto, 1.8 mi strip on the north bank of rio Guaimoreto between old bridge and opening of Laguna Guaimoreto 4.5 NE of Trujillo on old road to Castilla, 15°57'30"N, 85°54'30"W, 0 m, 10 July 1980, fl., J.G. Saunders 453 (MO); Trujillo, 1.8.mi strip on the north bank of rio Guaimoreto between old bridge and opening of Laguna Guaimoreto 4.5 mim, NE of Trijillo on old road to Castilla, 15°57'N, 85°54'W, 0 m, 19 June 1980, fl., J. Saunders 397 (MO). **NICARAGUA. Atlántico Sur**: Rio Pijibaye, 11°27'N, 83°54'W, 10–20 m, 18 Feb 1995, fl., fr., R. Rueda 3216 (MO). **Río San Juan**: Municipio de San Juan del Norte, Reserva Indio-Maíz, entre San Juan del Norte y la Finca de Chepelión, 50 m, 8 July 2002, fl., R. Rueda 16901 (MO). **PANAMA. Bocas del Toro**: Water Valley, vicinity of Chiriqui Lagoon, 23 Nov 1940, fl., H. von Wedel 1754 (MO). **Colón**: Chagres, Isthmus of Panama, 26 Mar 1850, fl., A. Fendler 206 (K); Portobello, ridge top, 1–3 miles W of Portobello, 7 Sept 1971, fl., A.H. Gentry 1766 (MO); premontane wet forest along Road S1 as it climbis the hill 1 Km SE of Camp Pina, 6 km WNW of Gatun Dam, 125 m, 21 Dec 1973, fl., M. Nee 8948 (MO); Western most part of province, site of proposed copper mine (INMET), Tailings Area, lowland forest on steep slopes, 8°53'50"N, 80°39'44"W, 40 m, 15 Apr 2009, fl., G. McPherson 20983 (MO). **Darién**: Rio Cupe, Rio Tuira between Boca de Cupe and mouth of Rio Pucro, 7°54'N, 77°30'W, 0 m, 12 Jan 1975, fl., A.H. Gentry 13528 (MO); Yavisa, Rio Chucunaque, 0–1 hour above Yaviza, near sea level, 8°10'48.0"N, 77°40'48.0"W, 0 m, 8 Jan 1975, fl., A.H. Gentry 13477 (MO). **Panama**: Barro Colorado Island, Canal Area, cove north of dock, 2 July 1970, fr., T.B. Croat 11085 (MO); Barro Colorado Island, Canal Zone, shore line N of Smithsonian Laboratory Habour towards Salud Point, 9. 16°, menos 79. 84°, 28 Feb 1964, fl., F. Ehrendorfer 6400-22 (WU); *Ibid.*, Shoreline, 24 Jan 1968, fl., fr., J.D. Dwyer 8450 (F); *Ibid.*, tip of Pearson Trail Peninsula S & W to 3rd large cove, 09°10'07"N, 79°51'31"W, 0–5 m, 7 May 1968, fr., T.B. Croat 5406 (MO). **BOLIVIA. Beni**: Riberlata, ca. 3 km SW of Riberalta on road to Hamburgo (crossing of Río Beni), várzea forest, heavily disturbed, 11°01'48.0"S, 66°06'00.0"W, 230 m, 20 Sept 1981, fl., J.C. Solomon 6349 (MO); Vaca Díez, Cachuela Esperanza Río Beni, 12 Sept 1985, fl., M. Moraes 550 (MO). **BRAZIL.** s.loc., s.d., fl., s.inf. s.n. (B). **Acre**: Porto Walter, Along Rio Juruá-Mirim, ca. 3 hrs by boat, above its mouth at Rio Juruá, 11 Nov 2001, fl., P. G. Delprete 7688 (NY). **Amapá**: Macapá, canteiro do Museu Macapá, 30 June 1981, fl., Verônica 17 (RBR); Rio Araguari, 1°10'48.0"N, 52°07'48.0"W, 22 July 1951, fr., R.L. Fróes 27540 (IAN, MO); Rio Calcoene, 16 Nov 1901, fl., W.A. Ducke s.n. (K, MO); Rio Puchacá afluente do Vila Nova, 15 Feb 1961, fl., A.G. Andrade 855 (R). **Amazonas**: Anavilhanas, Rio Negro, May 1980, fl., M. Goulding 66a (MG, MO); *Ibid.*, s.d., fl., M. Goulding 2120 (MG, MO); Autaz-Mirim, igapó do Curi, 20 Mar 1973, fl., fr., A.A. Loureiro INPA37554 (INPA); *Ibid.*, Lago do Purupuru, igapó, 17 Mar 1973, fl., fr., A.A. Loureiro s.n. (INPA); Careiro, Lago do Castanho, igapó, 7 June 1972, fr., M. Honda INPA36004 (INPA); Itaubarana, Rio Purus region, Rio Ipixuna, 15 km downstream from Itaubarana (30 km from Tapaua), igapó, 5°38'0"S, 63°10'0"W, 19 Jan 1986, fl., G. Gottsberger 115-19186 (MO); Itaubarana, Rio Purus region, Rio Ipixuna, 15 km downstream from Itaubarana (30 km from Tapaua), igapo, 5°38'0"S, 63°10'0"W, 23 Jan 1986, fl., G. Gottsberger 16-23186 (MO); Janauacá, Lago do Castanho, 7 June 1972, fr., M. Honda INPA36004 (INPA); Manaus, bank of Rio Negro and Rio Amazonas near Manaus, 4 Apr 1974, fl., fr., A.H. Gentry 11191 (MO); *Ibid.*, Campus do INPA, Séde do INPA, estrada do Aleixo, km 3, capoeira, solo argiloso, 5 Aug 1973, fl., P.N. Conceição 5 (INPA); *Ibid.*, estrada do Igarapé do Mariano, capoeira fechada, 23 Apr 1958, fl., fr., J.C. Almeida INPA6383 (INPA); *Ibid.*, Igarapé do Binda, 19 Jan 1955, fl., J.C. Chagas 606 (K); *Ibid.*, terra úmida, 2°18'36.0"S, 60°04'48.0"W, 19 Jan 1955, fl., J.C. Almeida INPA606 (INPA); Novo Airão, margem do Rio Negro, mata de igapó, 01°54'21.0"S, 61°20'08.9"W, 21 m, 12 May 2015, fl., M. Beyer 324 (SPF); *Ibid.*, 1°40'07.0"S, 61°25'00.1"W, 13 m, 12 May 2015, fl., M. Beyer 332 (SPF); *Ibid.*, Estação Ecológica Anavilhanas, 2°31'48.0"S, 60°50'24.0"W, 5 Feb 2007, fl., L.G. Lohmann 836 (SPF); Presidente Figueiredo (entorno), beira do Rio Uatumã, abaixo do Ramal da Morena, 1°00'S, 59°00'W, 24 Feb 2007, fl., C.E. Zartman 6349 (INPA, SPF); *Ibid.*, beira do Rio Uatumã, abaixo do ramal da Morena, 24 Feb 2007, fr., C.E. Zartman 6333 (INPA); *Ibid.*, Balbina, 2°01'48.0"S, 60°01'12.0"W, 13 Aug 2008, fl., F.F. Melo 532 (INPA); Rio Ituxi, Boca du Curuquete, Rio Purus, 10 July 1971, fl., G.T. Prance 14036 (INPA); Rio Negro between Rio Quinini and Moreira, sandy river bank, 13 Oct 1971, fl., G.T. Prance 15178 (INPA); *Ibid.*, Parana do Jacaré, 2°01'48.0"S, 60°01'12.0"W, 24 June 1992, fl., S.A. Mori 22470 (MO); São Francisco, Rio Negro, Paraná do Camanaú até Ponta do Canta Galo, 1°41'24.0"S, 61°16'12.0"W, 25 Apr 1973, fl., fr., M.F. Silva 1092 (INPA); Solimões, boca do Tefé, capoeira, 27 Sept 1904, fl., A. Ducke MG6821 (INPA); Tefé, Lago Tefé, northwest shore, igapó habitat, sandy, flooded lakeshor, 11 Dec 1982, fl., T. Plowman INPA126243 (INPA); Xiborema, solo argiloso, mata de várzea, 1 Jan 1957, fl., L.F. Coêlho s.n. (INPA). **Maranhão**: Lago Verde, fazenda São Francisco, 11Km N of Km 337 of BR 316, forest with Orbignya Palm, 4°0'S, 44°56'W, 25 Sept 1980, fr., D.C. Daly D264 (MG); Palmeirândia, 17 Dec 2006, fl., C.M. Vieira 72 (IAN). **Mato Grosso**: Colíder, canteiro de obras da UHE Colíder, terra firme, 10°58’ 48.0"S, 55°46'12.0"W, 258 m, 6 June 2011, fl., C.R.A. Soares 3594 (HERBAM, NX); near Tabajara, upper Machado River region, Nov 1931, fl., B.A. Krukoff 1517 (K, NY, P). **Pará**: Aguá, Rio Irucú, mata de várzea, 1992, fl., fr., U.R. Maciel 1971 (MG); *Ibid.*, Rio Marajozinho, mata de várzea, 1992, fl., U.R. Maciel 1793 (MG), U.R. Maciel 1823 (MG); Almeirim, Distrito de Monte Dourado, coletas ao longo do Rio Jari, 0°51'00"S, 52°32'00"W, 68 m, 4 July 2010, fl., R.C. Forzza 5994 (RB); Altamira, Rodovia Transamazônica (BR-230), margem do Rio Xingú, antes da travessia da Balsa, lado esquerdo da rovovia, sentido Altamira - Marabá, 03°07'34.4"S, 51°42'03.2"W, 5 m, 30 Nov 2005, fl., R.G. Udulutsch 2708 (HRCB, MBM, SPF, UNESPRC); Aveiro, Flona do Tapajós, Rio Cupari, 12 May 2011, fl., M.A. Braga 77 (RB); Belém, Campus of IPEAN, 6 Dec 1974, fl., A.H. Gentry 13075 (MO); Belterra, caminho para Pindobal, 29 Oct 1947, fl., G.A. Black 47-1861 (IAN, K); Chaves, Ilha Mexiana, Faz. Nazareth, 18 Sept 1901, fl., M. Guedes s.n. (MO); estrada entre S. Miguel e Río Caracuru, varzea, capoeira, 17 Jan 1969, fl., N.T. Silva 1654 (K, MO); Furo Macujubirm, 30 Aug 1901, fl., M. Guedes s.n. (MO); Ilha de Marajó, Rio Gipuru, 00°15'S, 50°30'W, 24 Oct 1987, fl., H.T. Beck 178 (F, MO); Melgaco, na Ilha do Marajó, Río Mapari, afluente do Río Tajapuru, 29 Nov 1991, NA, G. Santos 226 (MO); Óbidos, capoeira, 5 Aug 1902, st., W.A. Ducke s.n. (K, MO); Ourém, capoeira, 4 Dec 1903, fl., R.S. Rorb s.n. (MG); Paraupebas, Reserva Biologica da Serra dos Carajás, Companhia Vale do Río Doce, área da planta piloto, mina de exploracao de ferro-N4, 500 m, 20 Nov 1991, fr., G. Santos 183 (G, MO); Piriá, Bankof Rio Piria, N of km 90, 28 Oct 1965, fl., G.T. Prance 1736 (IAN, K, MO); Rio Mojú, 1 June 1954, fl., G.A. Black 54-16283 (K); Río Tocentius, reg. de S. Joazuim de Itaquara, 18 Dec 1960, fr., E. Oliveira 1243 (IAN, MO); Santarém, 1877-78, fl., M. Jobert 857 (P); São Sebastião da Boa Vista, estrada de acesso a Vila Cocal, 2 Sept 1992, fl., C.A. Santos 31 (MG); Senador Jose Porfirio (Sozel), margem direita do Rio Xingu, 02°34'00"S, 51°55'00"W, 3 Dec 1991, fr., G. Santos 287 (MO); Tucuruí, Breu Branco, igapó às margens do rio Tocantis, 14 Oct 1983, fl., J. Revilla 8681 (NY, SPF); *Ibid.*, Breu Branco, margem do rio Tocantis, 12 Sept 1983, fl., F.E. Miranda 576 (NY); Vitória do Xingu, Rio Xingu, Sítio Pimental, 2°52'48.0"S, 52°00'36.0"W, 15 Jan 2012, fl., C.S. Rosario s.n. (MBM). **Rondônia**: Costa Marques, às margens do rio Caltário, 28 Oct 1996, fr., L.C.B. Lobato 2398 (MG); Pacáas Novos, Rio Pacáas Novos, 3 Aug 1968, fl., G.T. Prance 6759 (INPA, K, MO, R). **Roraima**: Caracaraí, Rio Branco, 0°56'46.4"S, 61°52'32"W, 36 m, 15 May 2015, fl., A. Frazão 149 (SPF); *Ibid.*, 0°36'14"N, 61°35'43"W, 50 m, 20 Mar 2012, fl., G. Martinelli 17395 (RB); *Ibid.*, paraná abalaô, 0°56'49"N, 60°58'14"W, 34 m, 27 Mar 2012, fl., fr., M. Nadruz 2647 (RB); *Ibid.*, próximo ao encontro com o Rio Negro, 1°22'24.5'S, 61°51'59.7"W, 34 m, 16 May 2015, fl., A. Frazão 153 (SPF); *Ibid.*, Parque Nacional do Viruá, margem do Rio Branco, igapó, 01°40'29.0"N, 61°11'24.6"W, 42 m, 26 Sept 2014, fl., J.N.C. Francisco 41 (SPF); *Ibid.*, próximo da sede do Parque, floresta de terra firme, 01°29'23.3"N, 61°00'09.1"W, 68 m, 24 Sept 2014, fl., J.N.C. Francisco 40 (SPF); *Ibid.*, 01°29'24.9"N, 61°00'11.4"W, 67 m, 24 Sept 2014, fl., J.N.C. Francisco 39 (SPF); Igarapé Água Boa, Río Mucajai between Pratinha and Río Apiau, 22 Jan 1967, fl., G.T. Prance 4012 (INPA, K, MO, R, US);Rorainópolis, Rio Branco, 00°43'46.3"S, 61°51'24.0"W, 32 m, 14 May 2015, fl., V. Thode 424 (SPF); *Ibid.*, Ponto 11, 0°56'24.0"S, 61°50'24.0"W, 22 m, 14 May 2015, fl., fr., A. Frazão 136 (SPF); *Ibid.*, boca do Rio Branco com o Rio Negro, 1°23'8"S, 61°52'46"W, 40 m, 25 Apr 2014, fl., R.C. Forzza 8094 (RB); *Ibid.*, 1°23'8"S, 61°52'46"W, 40 m, 25 July 2014, fl., R.C. Forzza 8113 (RB); *Ibid.*, mata de igapó com interferência da água branca do rio Branco, 1°23'0.2"S, 61°51'6"W, 35 m, 13 May 2015, fl., B.M. Gomes 648 (SPF); *Ibid.*, em direção à Caracaraí, mata de igapó, 1°12'12.7"S, 61°50'37.3"W, 34 m, 13 May 2015, fr., B.M. Gomes 651 (SPF); *Ibid.*, ao encontro dos rio Branco com o Negro, mata de igapó com influência das águas do rio Negro, 1°5'46.5"S, 61°52'53"W, 28 m, 15 May 2015, fl., B.M. Gomes 659 (SPF); *Ibid.*, mata de igapó, 1°14'42.7"S, 61°50'56.2’’W, 29 m, 16 May 2015, fl., B.M. Gomes 662 (SPF); Rorainópolis, Rio Negro, mata de igapó, 01°33'14.2"S, 61°30'27.8"W, 13 m, 12 May 2015, fl., M. Beyer 336 (SPF); *Ibid.*, 01°33'14.2"S, 62°30'27.8"W, 13 m, 12 May 2015, fl., M. Beyer 337 (SPF); *Ibid.*, 1°22'5.2"S, 61°45'55.3"W, 18 m, 13 May 2015, fl., B.M. Gomes 639 (SPF); *Ibid.*, Rio Xixuaú, floresta beirando pequenos igarapés, 0°48'22"S, 61°33'32"W, 5 Mar 2010, fl., M.J.G. Hopkins 1961 (INPA); *Ibid.*, Ilha da casa do Chris, 0°48'01"N, 61°33'29’’W, 25 m, 3 Feb 2011, fl., T. Marinho 208 (INPA). **COLOMBIA.** Llamos de Cumaral ad vedem Andim bogosensim orinocum versus, 386 m, Jan 1876, fl., L. Aruz 1035 (P). **Amazonas**: Araracuara, rocks along Rio Caqueta, Araracuara, 17 Jan 1989, fr., A.H. Gentry 64809 (MO). **Antioquia**: Chigorodo, Rio Leon 15 km W of Chigorodo, 07°45'N, 76°50'W, 100 m, 19 Mar 1962, fl., C. Feddema 1954 (MICH, MO, US); Necoclí, Reserva Indígena Cainán Nuevo, 76°46'W, 8°36"N, 2 m, Aug 1992, fl., L. Castaño 93a (HUA). **Bolívar**: La Raya, Achi, Inspeccion de la Raya, 8°19'48.0"N, 74°33'36.0"W, 30 m, 5 May 1987, fl., H.V. Cuadros 3601 (MO). **Chocó**: Boca del Togoroma, Bank of Quebrada Togoroma, 13 June 1944, fl., fr., E.P. Killip 39122 (COL, F, MO, US); Las Animas, Jequedo, 42 km W of Las Animas, E of Rio Pato on Pan American (under construction) W of Las Animas, 5°16'48.0"N, 76°36'36.0"W, 250 m, 11 Jan 1979, fl., A.H. Gentry 23990 (MO); Truando, cativo swamps along Rio Truando, 18 Jan 1974, fl., A.H. Gentry 9313 (MO). **FRENCH GUIANA.** Javanes de Mana, 1855, fl., Gusllet s.n. (P); s.loc., 15 Dec 1956, fl., fr., A. Lemée 11 (P); s.loc., 1845, fl., fr., M. Melinón 64 (P); s.loc., 1856, fl., s.inf. s.n. (P); s.loc., 8 May 1874, fl., M. Melinón 121 (P); s.loc., Jan 1900, fl., F. Geay 1861 (P). **Cayenne**: Camopi, Camopi River, env. 12 km en amout de Camopi, 16 Dec 1965, fl., R.A.A. Oldeman 1796 (P, MO); Mahury River, Crique Gabrielle, tributary of the Mahury River, across from Stoupan, 4°45'00.0"N, 52°18'36.0"W, 10 m, 18 Oct 1991, fl., S.A. Mori 22137 (MO, NY); Mana River, Awara, village Galibi sur la reiver S de l’estucire de la Mana, env. à 18 km de Mana, 26 Jan 1978, fl., A. Raynal-Roques 19920 (MO, P); Maroni River, 1861, fl., M. Melinón 201 (P); *Ibid.*, 1982, fl., M. Melinón 205 (K, P); Montagne de Kaw, Montagnes de Kaw, Auberge de Brousse des Cascades, savanna and forest edges at end of road, 4°34'48.0"N, 52°16'48.0"W, 140 m, 12 Sept 1987, fl., A. Weitzman 287 (MO); Rives de l’Oyapock, entre St George at Maripa, Mar 1968, fr., R.A.A. Oldeman B-1449 (MO); Rivière Counana, affluent de l’Orapu, Dégrad Counana, 23 Dec 1966, fr., R.A.A. Oldeman B-778 (P); pont sur la crique Kourouaie, RN2, Régina, 04°06'53"N, 52°03'37"W, 4 m, 21 Mar 2009, fl., O. Tostain 2664 (P, NY, US). **GUYANA.** Pomeroon river, Mora Island, Wakapoa, 27 Dec 1958, fl., V. Graham P232 (K); s.loc., s.d., fl., Senudeas s.n. (P); Madoony Creek, Jan 1889, fl., G.S. Jenman 20968 (K). **Cuyuni-Mazaruni**: Mazaruni Station, 28 Aug 1937, fl., N.Y. Sandwith 1226 (K); *Ibid.*, 29 Oct 1943, fl., Fanshawe 4155 (K). **Upper Demerara-Berbice**: Moraballi Creek near Bartica, Essequibo River, 15 Nov 1929, fl., N.Y. Sandwith 617 (K, MO). **Venezuela**: Beryen de L’orenoque, 1864, fl., R. Grosourdy 13 (P). **PERU. Amazonas**: Condorcanqui, Monte virgin, 1 km atras de la comunidad de Caterpiza, trocha de metayar, banda este de la Quebrada Caterpiza, Rio Santiago, 3°54'36.0"S, 77°42'00.0"W, 180 m, 30 Oct 1979, fl., V. Huashikat 1145 (MO). **Huánuco**: Pachitea, region of Pucallpa, western part of the Sira mountains and adjacent lowland, c 26 km of Puerto Inca, next to the junction of the Rio Pachitea and Rio Yuyapichis, biological field station Panguana, primary lowland rain forest with some xer, 9°36'36.0"S, 74°55'48.0"W, 260 m, 21 Sept 1988, fl., fr., W. Morawetz 11-21988 (MO) . **Loreto**: Boca del Rio Itaya, above Iquitos, 110 m, 17 Sept 1929, fl., E.P. Killip 29401 (F); Indiana, trail from Indiana on Rio Amazonas to Rio Napo, well drained upland forest on clay, 3°27'36.0"S, 73°00'00.0"W, 200 m, s.d., fl., A.H. Gentry 22205 (MO); Iquitos, Carretera de Picuruyacu, en terreno arenoso, 3°44'24.0"S, 73°14'24.0"W, 160 m, 23 Sept 1981, fl., Y.M. Rimachi 5716 (MO); Maynas, explorer’s inn tourist camp near Indiana on Rio Amazonas, seasonally inundated tahuampa forest, 3°30'S, 73°00'W, 120 m, 21 Feb 1988, fl., A.H. Gentry 61828 (MO); *Ibid.*, Mishana, (Rio Nanay), bosque secundario de mas de 20 años, 03°55'S, 73°35'W, 130 m, 25 July 1984, fl., R. Vásquez 5404 (MO); *Ibid.*, Pto. Almendras (Rio Nanay), bosque inundable estacional (tahuampa), 3°48'00.0"S, 73°24'36.0"W, 122 m, 7 Sept 1984, fl., R. Vásquez 5540 (MO); Requena, Sanangal, bosque secundario inundable (Tahuampa), 04°10'S, 73°20'W, 120 m, 8 Aug 1980, fl., R. Vásquez 347 (MO). **Madre de Dios**: Laguna Cocacocha, edge of Laguna Cocacocha 39 km SW of Pto Moldanado near confl of Rios La Torre & Tambopata, 17 Oct 1968, fl., fr., S.F. Smith 408 (MO, US); Manu, Puerto Maldonado, Los Amigos Biological Station, ca. 7km upriver from mouth of Rio Los Amigos, between Cocha Llena and Cocha Lobo, 12°34'12.0"S, 70°06'00.0"W, 270 m, 4 Nov 2001, fr., J.P. Janovec 2606 (SPF); Tambopata, Cusco Amazonico, 15 km ENE of Puerto Maldonado, 12°24'48.0"S, 69°04'48.0"W, 200 m, 17 Dec 1989, fr., A.H. Gentry 68887 (MO); *Ibid.*, explorer’s inn tourist camp at junction of Rios La Torre and Tambopata, swampy forest, 12°48'36.0"S, 69°42'36.0"W, 270 m, 28 July 1985, fl., A.H. Gentry 51536 (MO). **SURINAME.** Île Portal, 1888, fl., P. Sagot s.n. (P); Sipiwalini, Voltzberg Nature Reserve, Coppename River, 1-2 Km north of Foengoe Island, 4°44'N, 56°11'W, 40 m, 21 Feb 1999, fl., B. Hoffman 5362 (MO). **VENEZUELA.** Des bords de l’Orinoque, 27 Sept 1886, fl., M. Chaffanjon 336 (P). **Amazonas**: Boca Casiquiare, selvas pluviales en y los alrededores de la orilla del Rio Casiquiare, entre la boca y Isla de la Paloma, 18 Feb 1986, fl., B. Stergios 9001 (MO); Carinagua, Dept Atures, alrededores de Puerto Ayacucho (ca. 9 Km al S), bosque de galeria del Caño Carinagua, alrededor del puente de la carretera Pto. Ayacucho-Samariapo, 10 Jan 1978, fl., O. Huber 1406 (MO); Dept. Átures, Puerto Ayacucho, end of road from airport to Rio Orinoco, gallery forest along river, 4 Apr 1984, fl., T. Plowman 13473 (F); Puerto Ayacucho, seasonally inundated forest at edge of Raudales del Orinoco, behind Pto. Ayacucho airport, sandy beach and adjacent laja, 5°39'36.0"N, 67°39'36.0"W, 100 m, 3 Apr 1984, fl., A.H. Gentry 46267 (MO); Rio Casiquiare, entre Piedra Guachapita y Curimacare, 2°00'00.0"N, 66°19'48.0"W, 150 m, 16 Jan 1987, fl., B. Stergios 9778 (MO); Rio Orinoco, caño Morocoto below San Fernando de Atabapo, 03°40'41"N, 67°14'15"W, 26 Mar 1974, fl., A.H. Gentry 10943 (MO). **Apure**: La Ceiba, between Rio Borgue and El Jordan, 7 km E de la Ceiba, 16 km E del Jordan, 350 m, 6 Apr 1968, fl., J.A. Steyermark 101948 (K); locally frequent along Rio Cinaruco for 20 km above las Galeras de Cinaruco, 24 Jan 1956, fl., J.J. Wurdack s.n. (JBRJ, RB); San Fernando, mouth of Rio Arauca at Rio Orinoco, 7°24'N, 66°36'W, 35 m, 14 May 1977, fl., G. Davidse 13198 (MO). **Bolívar**: Dpto. de Atures, Territorio Federal Amazonas, bosque humedo del rio Cataniapo, cercano a la desembocadura con el rio Orinoco, 6°24'36.0"N, 67°24'36.0"W, 37 m, 15 Feb 1983, fl., A. Castillo 1604 (MO); Moitaco, Distrito Sucre, rebalse del Orinoco, Hato Curumutopo, 11 Sept 1963, fl., fr., G. Martino 18 (MO, NY); Rio Orinoco, frequent on rocky outcrops on Isla Sta. Elena, opposite mouth of Rio Pargueni, 80 m, 13 Dec 1955, fl., J.J. Wurdack 39860 (K); Rio Parhueña, near Rio Pargueña, 38–40 km N of Puerto Ayacucho, 6°21'00.0"N, 67°09'36.0"W, 100 m, 30 June 1975, fr., A.H. Gentry 14686 (US, MO). **Delta Amacuro**: Depto. Antonio Diaz, Cano Atoiba, 9°15'N, 60°57'W, 50 m, 19 Oct 1977, fl., J.A. Steyermark 114985 (MO); Depto. Antonio Diaz, Cano Joba-Suburu, 8°59'N, 61°00'W, 50 m, 21 Oct 1977, fl., fr., J.A. Steyermark 115147 (MO); Rio Amacuro, between Amacuro and mouth of Deadwater Creak Moat, 8°31'12.0"N, 60°28'12.0"W, 65 m, 7 Nov 1960, fr., J.A. Steyermark 87341 (MO); *Ibid.*, between Amacuro and mouth of Deadwater Creek Moat, 8°31'12.0"N, 60°28'12.0"W, 65 m, 7 Nov 1960, fl., J.A. Steyermark 87347 (MO).

### 5. Pachyptera linearis

Taxon classificationPlantaeLamialesBignoniaceae

Francisco & L.G.Lohmann sp. nov.

urn:lsid:ipni.org:names:60475860-2

Fig. 13

#### Type.

Colombia. Meta: Parque Nacional Natural Tinigua, Serrania Chamusa Centro de Investigación Primatologicas La Macarena, 120 m, Apr 1992, fr., P. Stevenson 403 (holotype: MO088944!; isotypes, COL000349706!, COAH21210, not seen).

#### Diagnosis.

*Pachyptera linearis* is similar to *Pachyptera kerere*, but can be distinguished by the linear and flattened capsule (vs. the fusiform and inflated capsule of *P. kerere*), inconspicuous longitudinal midline on each valve (vs. conspicuous and raised longitudinal midline on each valve of *P. kerere*) and thin, oblong and wingless seeds (vs. corky, irregularly circular, obcordate and wingless seeds of *P. kerere*).

#### Description.

*Liana*; stems solid, tetragonal (cylindrical in younger portions), with greyish striations, lenticellate; prophylls of axillary buds 3-seriated, flattened and ensiform. *Leaves* with blades discolorous or concolor, membranaceous to chartaceous, elliptic, obovate-lanceolate, asymmetric, apex acute, acuminate, base cordate, lateral blades 6.0–12.4 × 3.0–4.9 cm, apical blades 8.0–14.7 × 3.4–6.0 cm; petiole semi-cylindrical, 1.0–4.2 cm long, petiolules not puvinated, lateral petiolules 0.5–1.6 cm long, apical petiolules 1.5–3.5 cm long. *Inflorescence* a congested raceme, 0.6–1.2 cm long; pedicel ca. 0.8 cm long; bracts ca. 0.7 mm long; bracteoles cymbiform, ca. 0.7 mm. *Calyx* green, tubular, minutely 5-lobed, 0.9 × 0.8 cm. *Corolla* white, infundibuliform, ca. 6.7 cm long, ca. 1.4 cm of diameter at the tube mouth; lobes rounded, 0.8 × 0.9 cm. *Androecium* with the longer stamens ca. 32.0 mm long, the shorter stamens ca. 10.9 mm long, glabrous; anthers villous, included, thecae curved forward, 2.5 × 0.9 mm; pollen 3-colpate, microreticulate. *Gynoecium* 5.0–5.2 cm long; ovary 2.9–4.0 × 1.2 mm, cylindrical, not-sulcate, smooth, sparsely to moderately pubescent, with simple trichomes, sparsely lepidote, with glandular peltate trichomes, without patelliform glandular trichomes; stigma capitate or ovate, 1.7–2.8 × 1.7–2.8 mm; nectar disc 1.8 × 2.0 mm. *Capsule* linear, flattened, 19.0–35.0 × 2.2–2.4 cm, each valve with an inconspicuous longitudinal midline; seeds oblong, 4.0–7.0 × 1.5–1.8 cm, thin, not-corky, coriaceous to woody, striated, secondary sculpture regularly interrupted by lateral rays, wingless, with short membranaceous or chartaceous and hyaline wings.

**Figure 13.**
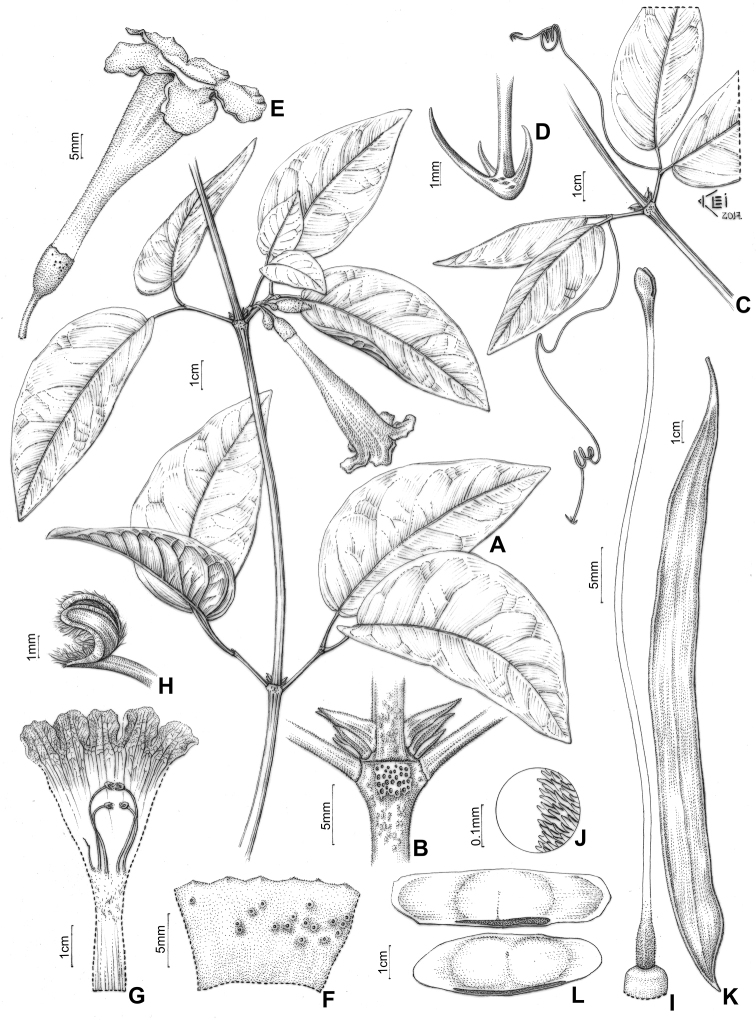
*Pachyptera linearis*. **A** Flowering branch **B** Interpetiolar region with ENFs and prophylls of axillary buds 3-seriated, flattened and ensiform **C** Branchlets with terminal leaflet replaced by trifid tendril (leaflet fells) **D** Trifid tentril **E** Flower **F** Calyx external view **G** Open flower showing the androecium with anthers united **H** Stamen with villous and curved thecae **I** Gynoecium **J** Ovary surface pubescent (J.J. Wurdack 41357, K) **K** Fruit flattened, with an inconspicuous longitudinal midline **L** Seeds wingless (P. Stevenson 403, MO).

#### Distribution.

**Figure 14.**
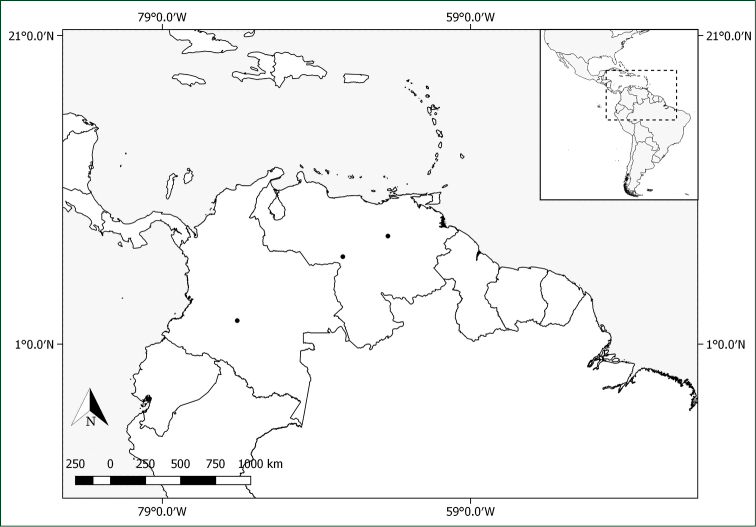
Distribution of *Pachyptera linearis*.

*Pachyptera linearis* is only known from wet forests of Venezuela (Apure, Bolívar) and Colombia (Meta). Fig. 14.

#### Phenology.

*Pachyptera linearis* flowers in January and fruits in April.

#### Etymology.

The epithet *linearis* makes reference to the linear fruit.

#### Nomenclatural note.

*Pachyptera linearis* is a new species described here based on new morphological and molecular phylogenetic data (Fig. 1; Francisco and Lohmann submitted). The best quality material was selected as the holotype.

#### Taxonomic comments.

*Pachyptera linearis* is characterised by white and infundibuliform corollas, with a sparsely pubescent and sparsely lepidote ovary. Furthermore, the capsule is linear, flattened, coriaceous to woody, with an inconspicuous longitudinal midline on the valves. The seeds are oblong, thin, coriaceous to woody, winged, with short membranaceous or coriaceous and hyaline wings. *Pachyptera linearis* shares the white infundibuliform flowers with its sister species *P. kerere* (Fig. 1). Nevertheless, *P. linearis* is easily distinguible from *P. kerere* by the fruit morphology.

#### Paratypes.

**VENEZUELA. Apure**: galeras del Cinaruco, Rio Cinaruco, 29 km above Las Galeras de Cinaruco, 80 m, 24 Jan 1956, fl., J.J. Wurdack 41357 (K, MO, RB, S, VEN). **Bolívar**: Moitaco, rebalse del Orinoco, Hato Curumutopo, 8°00'00.0"N, 64°21'36.0"W, 24 Apr 1991, fr., G. Martino 22 (MO).

### Doubtful and excluded names

Bignonia incarnata var. caribaea DC., Prodr. 9: 154. 1845. Type: Guadeloupe. s.loc., s.d., fl., F.L. L’Herminier s.n. (holotype, G-DC [G00133268]!) = *Bignonia aequinoctialis* L.

*Pachyptera alliacea* (Lam.) A.H. Gentry Brittonia 25(1): 236. 1973. Type: French Guiana. s.loc., s.d., fl., J.B.C.F. Aublet s.n. (holotype, P-AD [P00307351]!) = *Mansoa alliacea* (Lam.) A.H. Gentry

*Pachyptera dasyantha* DC. Prodr. 9: 176. 1845. Type: Brazil. Bahia: Rio São Francisco, s.d., fl., J.S. Blanchet 2903 (holotype, G-DC [G00133367]!, K not seen) = *Tanaecium pyramidatum* (Rich.) L.G. Lohmann

*Pachyptera hymenaea* (DC.) A.H. Gentry Brittonia 25(3): 236. 1973. Type: Brazil. Bahia, s.d., fl., J.S. Blanchet 1434 (holotype, G-DC [G00133196]!; isotype, P00481498!) = *Mansoa hymenaea* (DC.) A.H. Gentry

*Pachyptera parvifolia* A.H. Gentry Phytologia 26(6):447–450. 1973. Type: Colombia, Sur de Santander, vicinity of Puerto BerRío between carare and Magdalena Rivers, raizudo, large liana, flowers light purple, forest at about 200m, 22 Apr 1937, fl., O. Haught 2179 (holotype, MO100091!) = *Mansoa parvifolia* (A.H.Gentry) A.H. Gentry

*Pachyptera perrottetii* DC. Prodr. 9: 176. 1845. Type: French Guiana, s.loc., s.d., fl., G.S. Perrottet 2851 (holotype, G-DC [G00133301]!) = *Tanaecium pyramidatum* (Rich.) L.G. Lohmann

*Pachyptera puberula* DC. Prodr. 9: 175. 1845. Type: Brazil: Mato Grosso: close to Cuiabá, s.d., fr., S. Manso 105a (holotype, G-DC [G00133299]!) = *Dolichandra uncata* (Adrews) L.G. Lohmann

*Pachyptera standleyi* (Steyerm.) A.H. Gentry Brittonia 25(3): 236.1973. Type: Guatemala, Quetzaltenango, between Finca Pirineos and Finca Soledad, lower southern slopes of Volcán de Santa María, between Santa María de Jesús and Calahuaché, 1300–1400 m, 5 Jan 1940, J.A. Steyermark 33533 (holotype, F1054546!; isotype, F1054531!, F-1054543! , US00125753!) = *Mansoa standleyi* (Steyerm.) A.H. Gentry

*Pachyptera striata* DC. Prodr. 9: 176. 1845. Type: Brazil, São Paulo, s.d., P.W. Lund 783 (holotype, G-DC [G00133363]!) = *Tanaecium pyramidatum* (Rich.) L.G. Lohmann

*Pachyptera umbelliformis* DC. Prodr. 9: 175. 1845. Type: Brazil, São Paulo, s.d., fl., C.F.P. von Martius (syntype, M not seen; isosyntype, G-DC [G00133346]!), Brazil, Rio da Paraiba, Neuwied (syntype, M not seen) = *Tanaecium pyramidatum* (Rich.) L.G. Lohmann

*Pachyptera ventricosa* (A.H. Gentry) L.G. Lohmann Ann. Missouri Bot. Gard. 99(3): 456. 2014. *Mansoa ventricosa* A.H. Gentry Ann. Missouri Bot. Gard. 66 (4): 783. 1979 [1980]. Type: Brazil, Pará: along Belém-Brasilia hwy., Km 345, 9 Aug 1963, fl., B. Maguire et al. 56083 (holotype, MO2232816!; isotypes, COL000004276!, MG136673!, NY00328882!, US00289053!) = *Mansoa ventricosa* A.H. Gentry

## Supplementary Material

XML Treatment for Pachyptera

XML Treatment for Pachyptera aromatica

XML Treatment for Pachyptera erythraea

XML Treatment for Pachyptera incarnata

XML Treatment for Pachyptera kerere

XML Treatment for Pachyptera linearis

## Appendix 1

Vouchers used in analysis on scanning electron microscopy micrographs study.

***Pachyptera aromatica*** (Barb. Rodr.) L.G. Lohmann: **BRAZIL. Amazonas**: 2−5 km N of Manaus-Itacoatiara, road at km 79 near Río Preto da Eva, 24 Nov 1974, fl., A.H. Gentry 12832 (MO); Iranduba, estrada entre Novo Airão e Manacapurú, 2°54'46.6"S, 60°57'58.8"W, fl., L.H. Fonseca 327 (SPF); Manaus, outskirts of Manaus, road to INPA, boat landing, behind airport, 26 Nov 1974, fr., A.H. Gentry 12862 (MG).

***Pachyptera erythraea*** (Dugand) A.H. Gentry: **COLOMBIA. Antioquia**: Caucasia, along road to Nechi 24 km from Caucasia-Planeta Rica road, hacienda Costarica, margin of primary forest and trees remaining in cleared pasture, 8°03'36.0"N, 75°04'48.0"W, 60 m, 21 Mar 1987, fl., J.L. Zarucchi 4887 (K).

***Pachyptera incarnata*** (Aubl.) Francisco & L.G. Lohmann: **BRAZIL. Amazonas**: Presidente Figueiredo, Balbina, Rebio Uatumã, grade do PPBio, 6 Oct 2006, fr., J.R. Carvalho-Sobrinho 1078 (INPA). **Pará**: Belterra, Floresta Nacional do Tapajós, estrada para comunidade de Jamaraguá, km 72, 02°55'15.9"S, 55°01'39.4"W, 114 m, 16 Sept 2015, fl., J.N.C. Francisco 89 (SPF); Óbidos, lago Maria Teresa, floresta de várzea, 01°52'38.2"S, 55°35'27.4"W, 14 m, 23 Sept 2015, fr., J.N.C. Francisco 122 (SPF); Santarém, ramal próximo à Usina Hidrelétrica Curuá-Uma, solo areno argiloso, floresta de terra firme, 02°48'45.2"S, 54°18'08.8"W, 47 m, 19 Sept 2015, fl., J.N.C. Francisco 105 (SPF).

***Pachyptera kerere*** (Aubl.) Sandwith: **BRAZIL. Roraima**: Caracaraí, Parque Nacional do Viruá, margem do Rio Branco, igapó, 01°40'29.0"N, 61°11'24.6"W, 42 m, 26 Sept 2014, fl., J.N.C. Francisco 41 (SPF); Caracaraí, Parque Nacional do Viruá, próximo da sede do Parque, floresta de terra firme, 01°29'23.3"N, 61°00'09.1"W, 68 m, 24 Sept 2014, fl., J.N.C. Francisco 40 (SPF); Rorainópolis, Rio Branco, Ponto 11, 0°56'24.0"S, 61°50'24.0"W, 22 m, 14 May 2015, fl., fr., A. Frazão 136 (SPF).

***Pachyptera linearis*** J.N.C Francisco & L.G. Lohmann *new sp.*: **VENEZUELA. Apure**: galeras del Cinaruco, Rio Cinaruco, 29 km above Las Galeras de Cinaruco, 80 m, 24 Jan 1956, fl., J.J. Wurdack 41357 (K). **Bolivar**: Moitaco, rebalse del Orinoco, Hato Curumutopo, 8°00'00.0"N, 64°21'36.0"W, 24 Apr 1991, fr., G. Martino 22 (MO).

## Appendix 2

**Index to numbered collections**

Specimens are listed by collector in alphabetical order, followed by collector’s number. Collections by anonymous collectors without date or other identifying features are not listed here. Type specimens are shown in bold.

Aguilar, R. 818, 4824 (kerere). Almeida, J.C. INPA137, INPA1540, INPA1687, INPA5722 (aromatica); INPA606, INPA6383 (kerere). Amaral, I.L. 1248 (incarnata). Andrade, A.G. 855 (kerere). Árbocz, G.F. 4256 (incarnata). Aruz, L. 1035 (kerere). **Aublet, J.B.C.F. s.n. (BM000992379) (kerere)**. Beck, H.T. 178 (kerere). Beyer, M. 324, 332, 336, 337 (kerere). Black, G.A. 47-1861, 54-16283 (kerere); 48-3355 (incarnata); 52-14674 (aromatica). Braga, M.A. 77 (kerere). Brant, A.E. 2917 (kerere). Campbell, D.G. P22008 (incarnata). Campbell, E.J.F 95 (kerere). Carvalho-Sobrinho, J.R. 1078 (incarnata). Castaño, L. 93a (kerere). Castillo, A. 1604 (kerere). Chaffanjon, M. 336 (kerere). Chagas, J.C. 606 (kerere); INPA1701 (aromatica). Coêlho, L.F. INPA1731 (aromatica); s.n.(kerere). Conceição, P.N. 5 (kerere). Croat, T.B. 5406, 11085 (kerere). Cuadros, H.V. 3601 (kerere). Daly, D.C. D264 (kerere). Dardano 48-3092 (incarnata). Davidse, G. 13198, 32046 (kerere). Delprete, P.G. 7688 (kerere). Duarte, A.P. 7048 (aromatica). Ducke, A. 239, 22698a, 22698b, 35624, s.n. (R), s.n. (MO) (aromatica); s.n. (K, MO), MG6821, (kerere); 24091 (incarnata). Dwyer, J.D. 8450, 12737 (kerere). Ehrendorfer, F. 6400-22 (kerere). Evando 542 (incarnata). Fanshawe 4155 (kerere). Feddema, C. 1954 (kerere). Fendler, A. 206 (kerere). Fonseca, L.H. 327 (aromatica). Forzza, R.C. 5994, 8094, 8113 (kerere). Francisco, C.M. s.n. (MG)(aromatica). Francisco, J.N.C. 39, 40, 41 (kerere); 89, 103, 105, 121, 122, 130, 151 (incarnata). Frazão, A. 136, 149, 153 (kerere); 313 (aromatica). Fróes, R.L. 27540 (kerere). Geay, F. 1861 (kerere). Gentry, A.H. 1766, 9313, 10943, 11191, 13075, 13477, 13528, 14686, 22205, 23990, 46267, 51536, 61828, 64809, 68887 (kerere); 13027, 13323 (incarnata); 15369, 15372, 15402, 20050 (erythraea); 11201, 11207, 12778, 12815, 12832, 12862, 12888, 13022, 13056, 69107, 69308 (aromatica). Gerolamo, C.S. 9 (aromatica). Gomes, B.M. 639, 648, 651, 659, 662 (kerere). Gottsberger, G. 16-23186, 115-19186 (kerere). Goulding, M. 66a, 2120 (kerere). Graham, V. P232 (kerere). Grayum, M.H. 4411, 8032 (kerere). Grosourdy, R. 13 (kerere). Guedes, M. s.n. (MO)(kerere). Gusllet s.n. (P)(kerere). Herrera, G. 7551 (kerere). Hoffman, B. 5362 (kerere). Honda, M. INPA36004 (kerere). Hopkins, M.J.G. 1570, 1543, 1574 (aromatica); 1961 (kerere). Huashikat, V. 1145 (kerere). Huber, O. 1406 (kerere). Janovec, J.P. 2606 (kerere). Jenman, G.S. 20968 (kerere). Jobert, M. 857 (kerere). Kataoka, E. 349 (aromatica). Killip, E.P. 29401, 39122 (kerere). Knowles, O.H. 1106, 1120 (incarnata). Krukoff, B.A. 1517 (kerere); 6845, 12511 (aromatica). Labroy, M. 1906 (aromatica). Lemée, A. 11 (kerere). Lobato, L.C.B. 2398 (kerere); 3870 (incarnata). Lohmann, L.G. 28, 794 (aromatica); 836 (kerere). Loureiro, A.A. s.n. (INPA), INPA37554 (kerere). Luís, s.n. (MG)(aromatica). Maciel, U.R. 1793, 1823, 1971 (kerere). Maguire, B. s.n. (INPA), 56679 (aromatica). Marinho, T. 208 (kerere). Martinelli, G. 17395 (kerere). Martino, G. 18 (kerere); 22 (linearis). McPherson, G. 20983 (kerere). Melinón, M. 64, 121, 201, 205 (kerere). Mello, F.C. s.n. (INPA)(aromatica). Melo, F.F. 532 (kerere). Miranda, F.E. 362 (incarnata); 576 (kerere). Moraes, M. 550 (kerere). Morawetz, W. 11-21988 (kerere). Mori, S.A. 15783 (incarnata); 22137, 22470 (kerere). Mota, C.D.A. 18, 26, INPA60703 (aromatica). Nadruz, M. 2647 (kerere). Nee, M.H. s.n. (INPA) (aromatica); 8948 (kerere). Neill, D.A. 4030 (kerere). Nogueira, A. 190 (aromatica). Oldeman, R.A.A. B-778, B-1449, 1796 (kerere). Oliveira, E. 1243 (kerere); 2790 (aromatica). Pereira-Silva, G. 8765 (incarnata). Perrottet, G.S. s.n. (P03578200)(kerere). Pires, J.M. 12075 (incarnata). Plowman, T. 13473, INPA126243 (kerere). Poiteau, M. s.n. (G00014105)(kerere). Prance, G.T. 1736, 4012, 6759, 14036, 15178 (kerere); 17773 (aromatica). Pyramo, R.V. PSACF_EX06147 (incarnata). Rabele, B. 1003 (aromatica). Ramiro Fonnegra, G. 2580 (erythraea). Ramos, J. 1011 (incarnata). Raynal-Roques, A. 19920 (kerere). Revilla, J. 8326, 8397 (incarnata); 8681 (kerere). Ribeiro, R.S. 78 (incarnata). Rimachi, Y.M. 5716 (kerere). Robles, R. 1121, 1391 (kerere). Rocha, J.B.P. 701 (incarnata). Rodrigues, W.A. 4578, 4693, 5476 (aromatica). **Romero-Castañeda, R. 4727, 4979 (erythraea)**. Rusby, H.H. s.n. (NY00313053), 1143 (kerere). Rosario, C.S. s.n. (MBM)(kerere). Rueda, R. 3216, 16901 (kerere). Sales, J. 1539 (incarnata). Sandwith, N.Y. 617; 1226 (kerere). Santos, C.A. 31 (kerere). Santos, G. 183, 226, 287 (kerere); 282 (incarnata). Sagot, P. (P)(kerere). Saunders, J.G. 397, 453 (kerere). Senudeas, s.n. (P)(kerere). Silva, B.R. PSACF_EX06201 (incarnata). Silva, M. 1024 (aromatica); 1604 (incarnata). Silva, M.F. 1092 (kerere). Silva, N.T. 1654 (kerere). Smith, S.F. 408 (kerere). Soares, C.R.A. 3594 (kerere). Solomon, J.C. 6349 (kerere). Souza, J.A. s.n. (INPA), INPA61048, INPA61920, INPA71827, INPA71828, INPA71829, INPA71832, INPA71839 (aromatica). Souza, V.C. 18583 (incarnata). Sucre, A. s.n. (R, RB), (RB)(aromatica). Standley, P.C. 73082 (kerere). Stergios, B. 9001, 9778 (kerere). **Stevenson, P. 403 (linearis)**. Steyermark, J.A. 39022, 87341, 87347, 101948, 114985, 115147 (kerere). Thode, V. 17, 424 (kerere). Tostain, O. 2664 (kerere). Tsugaru, S. B-690 (aromatica). Udulutsch, R.G. 2708 (kerere). Ule, U. 4217, 5217 (aromatica). Vásquez, R. 347, 5404, 5540 (kerere). Verônica 17 (kerere). Vieira, C.M. 72 (kerere). Wedel, H. von 1754 (kerere). Weir, M. 72 (erythraea). Weitzman, A. 287 (kerere). Williams, L. 18222 (incarnata). Woodworth, R.H. 363 (kerere). Wurdack, J.J. s.n. (JBRJ, RB), 39860 (kerere); 41357 (linearis). Zartman, C.E. 6333, 6349 (kerere). Zarucchi, J.L. 4862A, 4887 (erythraea).
